# Robotic Home-Based Rehabilitation Systems Design: From a Literature Review to a Conceptual Framework for Community-Based Remote Therapy During COVID-19 Pandemic

**DOI:** 10.3389/frobt.2021.612331

**Published:** 2021-06-22

**Authors:** Aylar Akbari, Faezeh Haghverd, Saeed Behbahani

**Affiliations:** Department of Mechanical Engineering, Isfahan University of Technology, Isfahan, Iran

**Keywords:** home based rehabilitation, stroke rehabilitaiton, COVID 19 pandemic, conceptual framework, rehabilitation robotics

## Abstract

During the COVID-19 pandemic, the higher susceptibility of post-stroke patients to infection calls for extra safety precautions. Despite the imposed restrictions, early neurorehabilitation cannot be postponed due to its paramount importance for improving motor and functional recovery chances. Utilizing accessible state-of-the-art technologies, home-based rehabilitation devices are proposed as a sustainable solution in the current crisis. In this paper, a comprehensive review on developed home-based rehabilitation technologies of the last 10 years (2011–2020), categorizing them into upper and lower limb devices and considering both commercialized and state-of-the-art realms. Mechatronic, control, and software aspects of the system are discussed to provide a classified roadmap for home-based systems development. Subsequently, a conceptual framework on the development of smart and intelligent community-based home rehabilitation systems based on novel mechatronic technologies is proposed. In this framework, each rehabilitation device acts as an agent in the network, using the internet of things (IoT) technologies, which facilitates learning from the recorded data of the other agents, as well as the tele-supervision of the treatment by an expert. The presented design paradigm based on the above-mentioned leading technologies could lead to the development of promising home rehabilitation systems, which encourage stroke survivors to engage in under-supervised or unsupervised therapeutic activities.

## Introduction

Coronavirus disease 2019 (COVID-19) is an infectious disease with serious public health risk declared a global pandemic by WHO on March 11, 2020. In the present time of the COVID-19 pandemic, the lives of individuals have been drastically affected due to the imposed restrictions such as social distancing, curfews, and travel restrictions. This situation has had a considerable impact on the lives of certain more vulnerable groups on a larger scale, namely people living with chronic diseases such as stroke. In a pooled data analysis published in the International Journal of Stroke (IJS), Aggarwal et al. emphasized that COVID-19 puts post-stroke patients at a greater risk of developing complications and death. In fact, the odds of severe COVID-19 infection increase by 2.5 times in patients with a history of cerebrovascular disease (Aggarwal et al., 2020). This calls for consideration of extra safety precautions for this vulnerable population to protect them from infected environments, namely strictly abiding by the quarantine rules and social distancing, as breaking them could prove fatal (Markus and Brainin, 2020).

Stroke is accounted one of the dominant causes of severe and long-term disability. It leads to a total or partial loss of aptitude to trigger muscle activation to perform any activity (Borboni et al., 2016). The broad spectrum of induced disabilities includes a reduced range of motion (ROM), strength of the affected limb, and abnormal inter-joint coordination (Chen J. et al., 2017). The sensitive/motor deficits impede the performance of activities of daily living (ADLs), such as reaching, grasping, and lifting objects, as well as walking, etc., in the surviving individuals (Chen J. et al., 2017; Heung et al., 2019). Patients must be subjected to rehabilitative treatment to help them regain the ability to perform daily activities independently. As Martinez-Martin et al. stated, “Rehabilitation can be defined as the step-by-step process designed to reduce disability and to optimize functioning in individuals with health conditions, enabling them to better interact with their environment.” (Martinez-Martin and Cazorla, 2019). In the post-stroke rehabilitation process, the period between the first and the sixth months after stroke, known as the post-stroke sensitive period, has been proved to bear the maximum recovery impact, both spontaneous and intervention-mediated. Indeed, according to the statistics presented in Krakauer et al. article, during the first four weeks of rehabilitation, failure of reaching an arm Fugl-Meyer score of at least 11 would indicate only a 6% possibility of regaining dexterity at six months (Krakauer 2006). Regarding the aforementioned issues, in the current restrictive climate of COVID-19, the crucial question to be addressed for post-stroke patients would be how to use this critical and limited window of time to achieve the best possible recovery.

Robot-mediated therapy for post-stroke rehabilitation offers highly repetitive, high-dosage, and high-intensity alternatives, while reducing labor intensity and the manual burden on therapists. Hence, myriads of studies have focused on exploring robotics technologies for post-stroke rehabilitation. An increasing amount of research has investigated the efficiency of different types of robotic rehabilitation systems and found that these interventions can effectively complement conventional physical therapy, e.g., Mehrholz et al. and Bertani et al. investigated the effects of robot-assisted gait and upper-limb training, respectively, and both concluded that using robotic technologies positively affects post-stroke recovery (Bertani et al., 2017; Mehrholz et al., 2020). To tackle the recent rising issues associated with restrictions caused by the pandemic, there is a need to speed up the process of providing autonomous and affordable care that can be transferred out of inpatient or out-patient facilities into home environments. This study reviews the existing home-based robotic rehabilitation interventions and proposes reliable concepts that can be used to confront the discussed problems.

Home-based rehabilitation systems can be considered viable options capable of promoting care delivery while adhering to physical distancing measures and reducing the potential exposure to the infectious virus along with protecting vulnerable stroke survivors. Besides, even prior to this pandemic, the demand for home-based rehabilitation far exceeded its availability, further emphasizing the importance of this form of rehabilitation as a sustainable solution. According to WHO, demand for rehabilitation is approximately ten times that of the capacity of the service that the current healthcare system can provide, in terms of both rehabilitation professionals and rehabilitative tools (Gupta et al., 2011; World Health Organization, 2017). This poses a clear priority on further extension of home-based rehabilitation, which could prove to be a better alternative than conventional care by maintaining physical distancing and ameliorating the saturated health service.

Home-based systems offer a platform for unsupervised or under-supervised therapy, in which the need for the physical presence of a therapist is reduced. Rehabilitative treatments need to be intensive with long duration to improve functional outcomes and motor recovery (Bütefisch et al., 1995). Compared to clinical therapy, home therapy potentially augments standard care and enables consistent treatment by increasing the frequency and duration of training sessions. Performing rehabilitation at home provides patients with a comfortable setting. It gives them a sense of control of therapy as it reduces their reliance on external assistance (Chen et al., 2019). This can result in the patients demonstrating enhanced motivation and engagement (Borghese et al., 2013). In terms of the associated costs, home-based therapy reduces the expenses compared with clinical-based therapy; for example, in statistics provided by Housley et al., a saving of $2,352 (64.97%) was reported (Housley et al., 2016).

However, deployment in the unsupervised context of home-based rehabilitation poses risks to patients. Therapists perceive risks to patients regarding the training/acquisition of harmful movements when unsupervised at home—abnormal movement can be damaging or slow recovery (Borghese et al., 2014). Careful system design and deployment measures need to be taken, such as providing adequate feedback on proper task execution, to preclude these movements. On the other hand, in robotic medical devices the occurrence of errors that cannot be accounted for or predicted during device design leads to patient injuries and more severe incidents in some cases (Kim, 2020), e.g., crashes in device operability, both in hardware and software, and errors induced by contextual barriers in patients home environment. In clinical settings, in case of such incidents, the physical presence of healthcare professionals could mitigate the risk, yet such an option is not available at home.

Due to the benefits mentioned above, robot-mediated home therapy has gained attraction in recent years. Its feasibility has been evaluated through several studies using state-of-the-art home robots. The literature surveys indicated the feasibility of self-administered treatment at home using rehabilitation robots in terms of functional outputs, training duration, user acceptance, motivation, and safety concerns. Remotely supervised participants of these studies exhibited increased motivation and autonomy in completing the prescribed task with no adverse events or edema. They also self-reported increased mobility, improved mood, and an outlet for physical and mental tension and anxiety (Sivan et al., 2014; Nijenhuis et al., 2015; Cherry et al., 2017; Bernocchi et al., 2018; Catalan et al., 2018). Catalan et al. compared the performance of a commercialized clinical upper-limb rehabilitation device and its newly developed home-based counterpart, which showed that functional outcomes of treatment are similar for home users and clinic patients (Catalan et al., 2018). This suggests that, although both groups would reach the task’s goals similarly in terms of session numbers, due to the higher frequency of home-based therapy, users are able to master tasks in a shorter timespan (Godlove et al., 2019).

This article is intended to be used as a general guideline for developing robotic home-based rehabilitation systems. For this purpose, first, the authors conducted a comprehensive review on developed home-based rehabilitation technologies of the last 10 years (2011–2020), categorizing them into upper and lower limb devices and considering both commercialized and state-of-the-art realms. The literature review analyzes and synthesizes the current knowledge of home-based robotic systems. This aims to provide a categorized comparison among reviewed literature leading to a classified roadmap to help guide current research and propose recommendations for advancing research development in this field. By addressing current challenges and shortcomings, three main aspects are considered in the proposed design paradigm, i.e., mechatronics, control, and user interface. While existing reviews take a generalized approach on home-based rehabilitation solutions—e.g., Chen et al. provided a systematic review based on the utilized technology types (Chen et al., 2019)—we focus and expand on robotic devices. In contrast to solutions relying solely on VR and game-based technologies, this paper addresses robotics-based technologies to cover the need for a significantly wider range of post-stroke patients, including patients who require external assistance reflecting the therapist’s role in unsupervised settings. In the end, a conceptual community-based robotic rehabilitation framework, offering smart rehabilitation, is also introduced.

## Home-Based Rehabilitation Systems

Over the last decade, researchers have been addressing existing challenges and requirements to design and develop rehabilitation robots suitable for home therapy. As a result, many at-home rehabilitation devices have been designed within the research realm, and some have been commercialized (Tables 1–4). In the following subsections, a comprehensive review of developed home-based rehabilitation technologies of the last ten years (2011–2020) is presented.

**TABLE 1:** Summary of state-of-the-art robotic systems for home-based upper-limb rehabilitation.

Device	Main features and drawbacks	Control strategy	DOF	Supported movements	Weight (kg)	Stroke severity	Outcome measures	Time after stroke
HandSOME Brokaw et al. (2011); Chen and Lum (2016b); Chen et al. (2017a)	Only assists with extension; adjustable hard stops to limit ROM; assists with hand opening, grasp, grip, pinch, and gross movements	Extension passive assistance	11-Passive	5 fingers E	0.22 (version 1) and 0.128 (version 2)	Moderate to severe	FM; MAS; MAL; ARAT	>6 months after stroke
HandMATE Sandison et al. (2020)	Customizable 3D printed components; both manual and automated calibration sequence options for a facilitated home-use; using force sensitive resistors (FSR) for intention detection; android app with 4 customized game	Passive assistance; triggered passive assistance	11-(One actuator for each finger)	5 fingers FE	0.34	Moderate to severe	N/A	Chronic
X-glove Fischer et al. (2016); Ghassemi et al. (2018)	Facilitated two-component donning; custom GUI; multi-user VR exercises; haptic feedback	Passive assistance; partial assistance; resistance	N/A	5 fingers E (assisted)/F (resisted)	N/A	Severe	CMSA-H; FMUE; ARAT; CAHAI-9 GWMFT-func; GWMFT-time; EXT; FMUE; GS; LPS (N); PPS; MMAS; MAL QOM	Subacute phase
My-HERO Yurkewich et al. (2020)	EMG-based intention detection; automated calibration	Triggered passive assistance	2 actuators	5 fingers FE	0.377	Severe	FMA-UE; FMA-Hand; CAHAI-13	>6 months post stroke
HERO Yurkewich et al. (2019)	Ease of donning/doffing (3/1 minutes with assistance)	Passive assistance	1 actuator	5 fingers FE	0.192	Broad range of severe hand impairments	MMAS; MTS; BBT; CAHAI	Acute and chronic
IOTA Aubin et al. (2013)	Targeting pediatric population; portable control box	Passive assistance; triggered passive assistance	2	Thumb FE; thumb add-abduction	0.23	N/A	N/A	N/A
Grasping rehabilitation device Park et al. (2013)	Pressure sensors for intention detection	Passive assistance; triggered passive assistance; partial assistance	2 actuators	N/A	N/A	N/A	N/A	N/A
Vanderbilt Gasser et al. (2017)	Bidirectional under-actuated tendon system; simultaneous fingers actuation; adjustable thumb design	Passive assistance	1-Active	4 fingers FE	0.4	N/A	N/A	N/A
WearME Zhou et al. (2019)	Performing resistive motion tasks	Resistance	3 actuators	Wrist FE; finger opposition	0.5	N/A	N/A	Chronic
Soft robotic exomusculature glove Delph et al. (2013)	sEMG-based intention detection; moving weight off hand by housing actuating components in a backpack	Partial assistance; resistance	5 actuators	5 fingers FE	6	N/A	N/A	N/A
BCI-controlled pneumatic glove Coffey et al. (2014)	Adaptable BCI-based controller	BCI-based passive assistance	1 actuator/15 DOF	Finger E	2	N/A	N/A	N/A
Soft robotic glove Polygerinos et al. (2015)	Size customizable; easy to don/doff; minimal ADL interference	Partial assistance	3 per finger	5 fingers FE	0.285	N/A	N/A	N/A
Anthropomorphic soft exosuit Klug et al. (2019)	sEMG-based intention detection; cascade control	Passive assistance; triggered passive assistance	4 actuators	4 fingers FE	0.13	N/A	N/A	N/A
Exo-glove (in et al., 2015)	Bend sensors for intention detection; extremely lightweight; soft tendon routing system designed for zero pretension of the tendons; introduced a slack prevention mechanism; pinch and grasping assistance/training	Triggered passive assistance	3 actuators/9 DOF	Index, middle finger and thumb FE	0.194	N/A	N/A	N/A
Wrist rehabilitation device Ambar et al. (2017)	Android-based game application; mouse-like joystick suitable for patients with grasping difficulties	N/A	3-Passive	Wrist FE; wrist add-abduction; forearm PS	N/A	N/A	N/A	N/A
e-Wrist Lambelet et al. (2020)	Easy one-handed donning/doffing	Partial assistance (AAN)	1-Active	Wrist FE	Distal module 0.238	N/A	N/A	Acute or subacute
					Proximal module 0.224			
SCRIPT Amirabdollahian et al. (2014); Nijenhuis et al. (2015); Ates et al. (2017)	Easy don/doffing; motivational game environment based on AD; a tele-robotic support platform with a reach and user-friendly user-interfaces	Triggered passive assistance	6-Passive (1-unactuated for thumb ab-adduction)	Wrist FE; 5 fingers FE	0.65	Mild to severe	FM; ARAT; MAL; SIS	Chronic (>6 months post-stroke)
Ambidexter Wai et al. (2018)	IoT-enabled; aesthetically appealing; 3D printed standard components; offers gamification	Passive assistance; partial assistance	3 DOF	Hand opening/closing; forearm PS; wrist FE	3	N/A	N/A	N/A
(HAL-SJ) Hyakutake et al. (2019)	Hybrid control algorithm, both for voluntary and autonomous control; interactive biofeedback	Passive assistance; triggered passive assistance	1-Active	Elbow FE	1.3	Mild	MAL; FMA-UE; ARAT	Chronic (>6 months post-stroke)
A home-based bilateral rehabilitation system Liu et al. (2020); Liu et al. (2018)	Bilateral rehabilitation; sEMG-based real-time stiffness control	Triggered passive assistance	1-Active	Elbow FE (active); shoulder add-abduction; shoulder FE; shoulder IE rotation	3.1	N/A	N/A	N/A
			3-Passive					
Soft robotic elbow sleeve Koh et al. (2017)	EMG for intention detection; motion capture system	Passive assistance; triggered passive assistance	1-Active	Elbow FE	N/A	N/A	N/A	N/A
SpringWear Chen and Lum (2018)	Spring operated	Passive assistance	5 DOF	Shoulder F; elbow E; forearm PS	1.2	N/A	Shoulder FE ROM; elbow FE ROM; forearm PS ROM	Chronic (>6 months post-stroke)
				Shoulder Horizontal abd-adduction; shoulder IE rotation				
PACER Alamdari and Krovi (2015)	Various control modes; parallel platform; 3D workspace	Passive assistance; partial assistance; resistance	N/A	Arm FE, add-abduction, medial/lateral rotation; elbow FE, forearm PS; wrist FE, and wrist add-abduction	N/A	N/A	N/A	N/A
HomeRehab Díaz et al. (2018); Catalan et al. (2018)	Cloud-based communication system; VR and gamification; wearable devices to record the physiological state of the user	Partial assistance (AAN); resistance	3-Active	Shoulder FE; shoulder Horizontal abd-adduction; elbow FE	7	N/A	N/A	N/A
ArmAssistJung et al. (2013); Perry et al. (2012); Tomić et al. (2017); Butler et al. (2017); Perry et al. (2016)	Portable device table; interactive games operating on a web-based platform; a global position and orientation detection mat; visual and auditory feedback; grasp and pinch exercises	Partial assistance (AAN)	4 DOF	Shoulder Horizontal abd-adduction; elbow FE; wrist PS	N/A	Moderate to severe	FMA-UE; BI; WMFT	Subacute
Active therapeutic device (ATD) Westerveld et al. (2014)	Arm weight support; 3D end-point manipulator; functional training of reaching tasks; inherently safe design	Passive assistance; resistance	N/A	N/A	25	N/A	N/A	N/A
PaRRo Washabaugh et al. (2019)	Inherently safe due to using passive actuators: eddy current brakes; adjustable resistance	Resistance	4 actuators	Planar 2D motions	N/A	N/A	N/A	N/A
RUPERT Zhang et al. (2011); Huang et al., 2016)	VR; GUI for remote supervision; size adjustable; built-in safety mechanism	Passive assistance; partial assistance	5 DOF	Shoulder FE; humeral IE rotation; elbow FE; forearm PS; wrist FE	N/A	Mild to moderate	FMA; WMFT	Chronic

**TABLE 2:** Summary of commercialized robotic systems for home-based upper-limb rehabilitation.

Device	Main features and drawbacks	Control strategy	DOF	Supported movements	Weight (kg)	Stroke severity	Outcome measures	Time after stroke
Saebo SaeboVR 2017; Adams et al. (2018); Doucet and Mettler (2018); Franck et al. (2019); Runnalls et al. (2019)	Fine motor skills, grasp, grip, pinch and release training; virtual world-based rehabilitation software for ADL; provides various additional treatment kits; arm weigh support; non-slip surface	N/A	N/A	Wrist FE and PS; 5 fingers FE	N/A	Mild to severe	WMFT; FMUE; ROM; ARAT; SIS	Subacute to chronic
WeReha Bellomo et al. (2020)	Simultaneously stimulating the cognitive aspect; fine movement training of the hand	N/A	N/A	Shoulder F; elbow FE; forearm PS; wrist PS	N/A	Mild to moderate	BBS; BI; FM; mRS	Chronic
SEM glove Osuagwu et al. (2020); Nilsson et al. (2012)	Pressure sensor for intention detection logic; tactile sensor and force sensor; power unit backpack	Partial assistance	3 Actuators/9 DOF	3 fingers F	0.7	N/A	N/A	N/A
IronHand Radder et al. (2019); Radder et al. (2018)	Pressure sensors for intension detection logic; assistive and therapeutic platform; motivating game-like environment	Partial assistance	9 DOF	3 or 5 fingers F	0.07	N/A	Use time; SUS; BBT; JTHFT; maximal pinch strength; maximal handgrip strength	N/A
Gloreha lite Bernocchi et al. (2018)	3D animations for simulated preview of the movement; dynamic arm supports; grasping, reaching and picking tasks; audio and visual feedback	Passive assistance; partial assistance	5-Active	5 fingers F	0.25	N/A	FIM; MAS	N/A
The motus hand Wolf et al. (2015); Butler et al. (2014); Linder et al. (2013a); Linder et al. (2013b)	Visual biofeedback; offers gamification; FDA class 1 device	Partial assistance	N/A	Wrist and fingers FE	control box: 6.37	Mild to severe	FMA; WMFT; ARAT; SIS; MAS	Subacute to chronic

**TABLE 3:** Summary of state-of-the-art robotic systems for home-based lower-limb rehabilitation.

Device	Main features and drawbacks	Control strategy	DOF	Supported movements	Weight (kg)	Stroke severity	Outcome measures	Time after stroke
Soft robotic sock Low et al. (2018); Low et al. (2019)	Not size adjustable; not customizable for treatment parameters; offers early bedside care; improves venous flow during inpatient usage (fabric-based form factor and silicone-rubber-based actuator design provide a greater chance of user acceptance due to its compliant nature)	Pneumatic-actuated assistance	N/A	Ankle PD	N/A	Severe	Ankle ROM	Acute and subacute
Wearable ankle rehabilitation robotic device Ren et al. (2017)	Size adjustable; progressive augmented real-time feedback; sensory stimulus	Triggered passive assistive; partially assistive (AAN); resistive (active assistive training; resistance training; passive stretching)	1-Active	Ankle PD	N/A	N/A	FMLE; MAS	Acute
Motorized ankle stretcher (MAS) Beom-Chan et al. (2017); Yoo et al. (2019)	Equipped with a customized software	Passive assistance	2-Active per leg	Ankle PD and eversion/inversion	N/A	N/A	Ankle ROM, gait parameters	At least 6 months post stroke
EMG-controlled Knee exoskeleton Lyu et al. (2019)	Provides multisensory feedback; EMG record for intention detection; customized gaming; ensures safety at software, electrical and mechanical levels; simplified setup process with setup time of around 1 minute	Triggered passive assistance	2-Active per leg	Hip FE; Knee FE	20 (total including the electronic components and battery),0.92 (the exoskeleton’s lower leg)	N/A	N/A	Tested on healthy subjects
Lower limb rehabilitation wheelchair system Chen et al. (2017b)	Equipped with a tele-doctor; patient interaction module including user interfaces for both patients and therapist; customized virtual reality game	Passive assistance	N/A	Knee FE-standing/lying (movement of back)	N/A	N/A	N/A	N/A
Lower limb rehabilitation robot (LLR-Ro) Feng et al., 2017)	Intelligent human-machine cooperative control system; mechanical, electrical, and software safety features	Passive assistance	3-Active per leg	Hip FE; Knee FE; ankle PD	N/A	N/A	N/A	Early phase of
i-Walker Morone et al., 2016)	Used either for training or as an assistive device	Partial assistance (AAN); progressive assistance	N/A	N/A	N/A	Mild to moderate	Tineti’s scal- MAS- BI-6MWT-10MWT	Subacute <90 days
Curara^®^ Mizukami et al. (2018); Tsukahara et al. (2017)	Non-exoskeletal structure	Synchronization-based assistance	2-Active for each leg	Hip FE; Knee FE	5.8	N/A	N/A	N/A
WA-H Moon et al. (2017); Sung et al. (2017)	Highly repetitive without fatigue; facilitates weight shifting by passive hip joints in coronal plane	Triggered assistance	2-Active per leg	Hip FE; Knee FE	25 (including battery)	Mild	FMAS-BBS-TUGT-SPPB	Mean 1 year after
GEMS-H Hwang-Jae Lee et al. (2019); su-Hyun Lee et al. (2020)	Flexible exoskeleton	Partial assistance (AAN)	1-Active (per leg)	Hip FE	2.8	Mild to moderate	FMA, BBS, K-FES	Chronic stroke
			2-Passive					
Eddi current braking knee brace Washabaugh and Krishnan (2018); Washabaugh et al., 2016)	Inherently safe due to utilization of passive actuators: eddy current brakes	Resistance	1-Passive	Knee FE	1.6	Mild to moderate	10MWT	Chronic

**TABLE 4:** Summary of commercialized robotic systems for home-based lower-limb rehabilitation.

Device	Main features and drawbacks	Control strategy	DOF	Supported movements	Weight (kg)	Stroke severity	Outcome measures	Time after stroke
Stride management assist (SMA) (by Honda) Buesing et al. (2015)	Single-charge operation time of 2 hours; not size adjustable but available 3 sizes: M, L and XL; only provides assistance in sagittal plane; one functional upper limb side is required for putting it on	Uses a mutual rhythm scheme to generates assist torques at specific instances during the gait cycle to regulate the user’s walking pattern	1-Active per leg	Hip FE	2.8	N/A	N/A	Chronic (more than one year)
ReWalk ReStore™ (by ReWalk robotics) Awad et al. (2020)	Offers an optional textile component for patients who require medio-lateral ankle support; a hand-held real-time monitoring device with a graphical interfaces; some adverse events involving pain in lower extremity and skin abrasions were reported by users	Partial assistance	1-Active per leg	Ankle plantarflexion/Dorsiflexion	5	N/A	10MWT	> two weeks
EksoNR (by ekso bionics) Carlan and singleorigin (2020)	Variable assistance modes	N/A	3-Active per leg	Hip FE and knee FE	N/A	N/A	N/A	N/A
HAL(by CYBERDYNE inc.) Nilsson et al. (2014); Kawamoto et al. (2013); Kawamoto et al. (2009)	Hybrid control algorithm, both for voluntary and autonomous control	Passive assistance; triggered passive assistance	3-Active per leg	Hip FE; Knee FE; ankle PD	14 (double leg model)	N/A	10MWT; BBS; TUGT; FM-LE; FES; BI; FIM	Early onset; chronic (more than a year)
AlterG bionic leg Wright et al. (2020); Iida et al. (2017); Stein et al. (2014)	Auditory and sensory feedback; easy and fast donning and doffing (approximately of 2 minutes); standardized overground functional tasks including sit to stand transfer	Partial assistance	1-Active	N/A	3.6	N/A	10MWT; 6MWT; TUGT; DGI; BBS; mRS;; accelerometry	Chronic stroke (>3 months since stroke diagnosis)

We conducted a literature review in the PubMed search engine. The search included the following terms: “Rehabilitation Robotics,” “Home-Based Rehabilitation,” and “Stroke Rehabilitation.” Secondary references and citations of the resultant articles were checked to further identify relevant literature and other available sources providing information on commercially available solutions. Reviewers screened the abstracts of the collected articles for extracting those satisfying the eligibility criteria. Studies were excluded if they were not implemented and/or did not demonstrate implementation potential in home settings, based on the criteria introduced by authors in the following subsections.

Approximately 70% of stroke patients experience impaired arm function (Intercollegiate Working Party for Stroke, 2012). Hemiparesis is prevalent in up to 88% of post-stroke patients, which mostly leads to gait and balance disorders, that even persists in almost one-third of patients even after rehabilitation interventions, leaving them with the inability in independent walking (Gresham et al., 1995; Duncan et al., 2005; Díaz et al., 2011; Morone et al., 2016). Lower-limb devices face critical challenges for home use, making the upper-limb the primary focus of early efforts in this field. The following subsections were set to cover both state-of-the-art and commercialized devices by categorizing based on the upper or lower targeted limb.

### Upper-Limb

#### State-of-the-Art: Systems in the Literature

Due to the significant results of active participation between patients and robots in functional improvement, the majority of current rehabilitative robots are equipped with electrical or pneumatic motors (Pehlivan et al., 2011). However, the inherent considerable weight of mounted motors precludes the device from being used during daily activities, since proximal arm weakness is prevalent among individuals with stroke. Thus, to allow hand rehabilitation during the performance of ADLs, HandSOME (Brokaw et al., 2011), a passive lightweight wearable device has been developed. The design is based on the concepts of patient-initiated repetitive tasks to rehabilitate and assist during ADL performance by increasing assistive torque with increasing extension angle. To help with opening the patient’s hand and assisting with finger and thumb extension movements, HandSOME uses a series of elastic cords, as springs, to apply extension torques to the finger joint. For safety precautions, adjustable hard stops are used to control the ROM. Studies demonstrated that HandSOME could benefit stroke patients with ROM improvement. While patients commented that the device was generally comfortable for use at home (Chen J. et al., 2017), there is a need to develop a remote communication system instead of weekly clinical visits. Yet, one of the disadvantages of the device is its inability of assistance level adaptation to patient performance. Addressing this issue, Sandison et al. built a wearable motorized hand exoskeleton, HandMATE, upon HandSOME. This device benefits from 3D printing technology for manufacturing the components; hence it can be optimally adjustable and customizable to fit the patients’ physiological parameters (Sandison et al., 2020). Combining hand orthosis with serious gaming, Ghasemi et al. have integrated eXtention Glove (X-Glove) actuated glove orthosis with a VR system to augment home-based hand therapy. For a facilitated donning, the device design allows for being put on by two separate components (Ghassemi et al., 2018). The glove provides both stretching therapy and extension assistance for each digit independently while allowing free movements and interaction with real-world objects (Fischer et al., 2016). Gasser et al. presented compact and lightweight hand exoskeleton Vanderbilt intended to facilitate ADLs for post-stroke hand paresis. The design includes an embedded system and onboard battery to provide a single degree of freedom (DOF) actuation that assists with both opening and closing of a power grasp (Gasser et al., 2017).

The Hand Extension Robot Orthosis (HERO) Glove is another wearable rehabilitation system that provides mechanical assistance to the index and middle fingers and thumb (Yurkewich et al., 2019). Linear actuators control the artificial tendons embedded into the batting glove’s fingers for finger extension and grip assistance. Yurkewich et al. proceeded with their research by introducing My-HERO, a battery-powered, myoelectric untethered robotic glove. The new glove benefits from forearm electromyography for sensing the user’s intent to grasp or release objects and provides assistance to all five fingers (Yurkewich et al., 2020). Addressing the pediatric disorders, such as stroke, causing thumb deformation, lightweight hand-mounted rehabilitation exoskeleton, the Isolated Orthosis for Thumb Actuation (IOTA), offers 2 degrees of freedom thumb rehabilitation at home while allowing for significant flexibility in the patient’s wrist (Aubin et al., 2013). The device is patient-specific and can be securely aligned and customized to the patient’s hand. The portable control box of the design enhances user freedom and allows rehabilitation exercises to be executed virtually anywhere. For recovering hand grasp function, Park et al. developed a robotic grasp rehabilitation device integrated with patient intention detection utilizing handle-embedded pressure sensors (Park et al., 2013). The device was designed for home use by being small in size, portable, and inexpensive. As one of the first wearable robots performing resistive training, Wearable Mechatronics-Enabled (WearME) glove was developed coupled with an associated control system for enabling the execution of functional resistive training. The soft-actuated cable-driven mechanism of the power actuation allows for applying resistive torque to the index finger, thumb, and wrist independently (Zhou et al., 2019).

Targeting individuals with functional grasp pathologies, Delph et al. developed an sEMG-based cable-driven soft robotic glove that can independently actuate all five fingers to any desired position between open and closed grip using position or force control and simultaneously regulate grip force using motor current (Delph et al., 2013). Coffey et al. integrated a soft pneumatic glove with a novel EEG-based BCI controller for an increased motor-neurorehabilitation during hand therapy at home (Coffey et al., 2014). In contrast to clinical BCI-mediated solutions, it is an inexpensive and simplified alternative for training the subject’s wrist and fingers at home together with a haptic feedback system. Polygerinos et al. presented a portable soft robotic glove that combines assistance with ADL and at-home rehabilitation (Polygerinos et al., 2015). Hydraulically actuated multi-segment soft actuators using elastomers with fiber reinforcements induce specific bending, twisting, and extending trajectories when pressurized. The soft actuators are able to replicate the finger and thumb motions suitable for many typical grasping motions, to match and support the range of motion of individual fingers. Furthermore, the device has an open palm design in which the actuators are mounted to the dorsal side of the hand. This provides an open-palm interface, potentially increasing user freedom as it does not impede object interaction. The entire compact system can be packaged into one portable waist belt pack that can be operated for several hours on a single battery charge. Gross and fine functional grasping abilities of the robotic glove in free-space and interaction with daily life objects were qualitatively evaluated on healthy subjects. Compared to other robotic rehabilitation devices, the soft robotic glove potentially increases independence, as it is lightweight and portable. An electrically actuated tendon-driven soft exosuit was developed for supporting and training hand’s grasp function. The device offers versatile rehabilitation exercises covering motion patterns, including both power and precision grip, on each independently actuated finger (Klug et al., 2019). The Exo-Glove, a soft wearable robot using a glove interface for hand and finger assistance, developed by In et al., employs a soft tendon routing system and an underactuated adaptive mechanism (In et al., 2015). Inspired by the human musculoskeletal system, Exo-Glove transmits the tension of the tendons routed around the index and middle finger at the palm and back sides to the body to induce flexion and extension motions of the fingers.

The majority of upper-limb devices are dedicated to wrist rehabilitation due to its importance for peoples’ daily work and life (Wang and Xu, 2019). By combining sensing technology with an interactive computer game, Ambar et al. aimed at developing a portable device for wrist rehabilitation (Ambar et al., 2017). To consider difficulties of stroke patients in firm grasping, the design was based on a single-person mouse-like joystick. The third DOF is considered for forearm pronation/supination, adding to standard flexion/extension and adduction-abduction movements for wrist rehabilitation. Clinical trials on healthy subjects using the device have shown task completion through a smooth recorded trajectory. In 2020, they also developed an android-based game application to enable patients to use the rehabilitation device at home or anywhere while making the therapy systematic and enjoyable (Ail et al., 2020). Lambelet et al. developed a fully portable sEMG-based force-controlled wrist exoskeleton offering extension/flexion assistance, eWrist. Given the prominence of the donning aspects of rehabilitation robots in unsupervised settings, the device was iteratively designed emphasizing attachment mechanism and distal weight reduction to enable one-hand and independent donning of the device (Lambelet et al., 2020).

**FIGURE 1:**
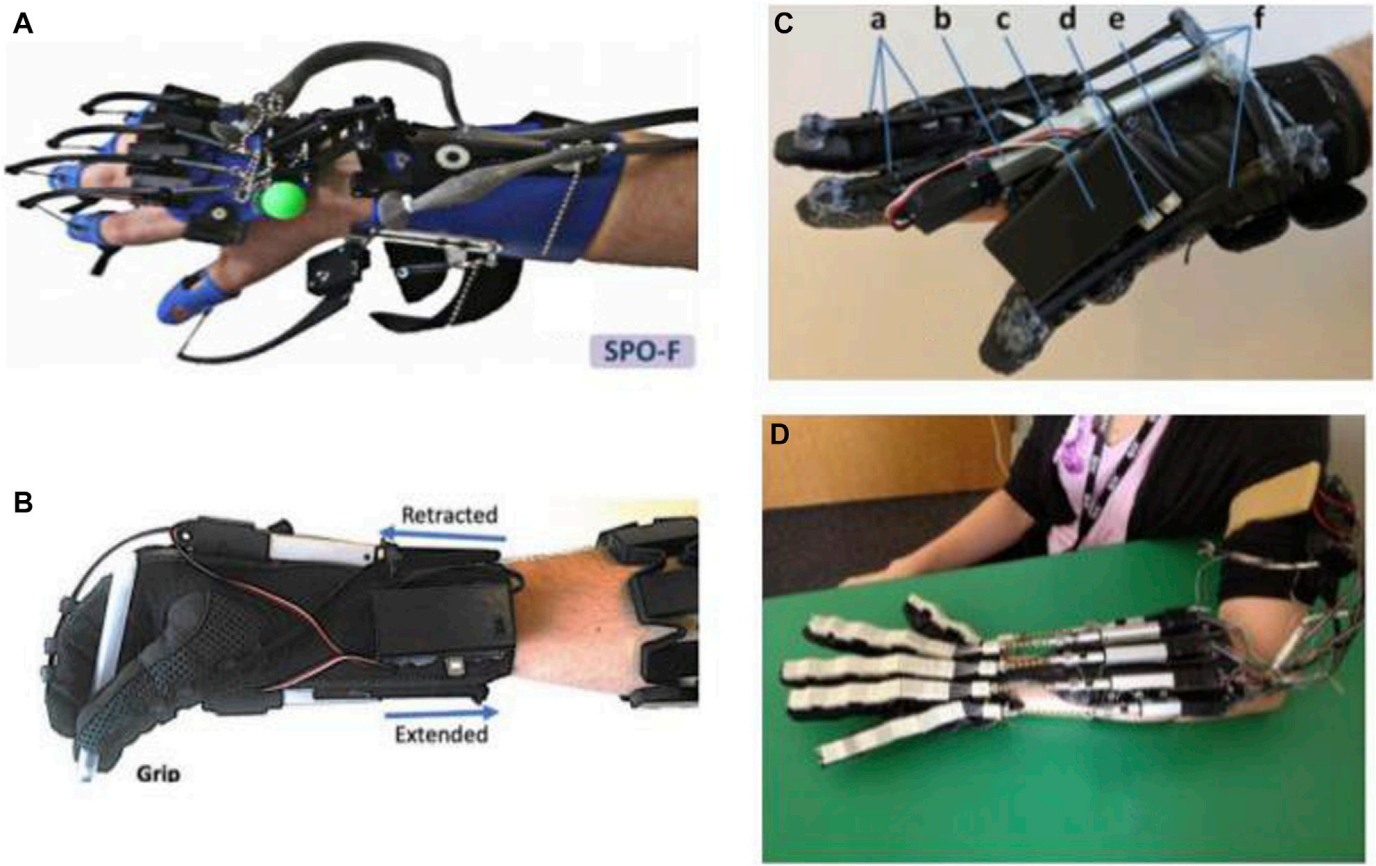
Robotic gloves and exoskeleton devices for home-based hand rehabilitation: **(A)** SCRIPT project (Ates et al., 2017), **(B)** My-HERO (Yurkewich et al., 2020), **(C)** HERO (Yurkewich et al., 2019), and **(D)** X-Glove (Triandafilou, 2014).

One of the first projects dedicated to enabling home rehabilitation is the SCRIPT project. SCRIPT project—Supervised Care, and Rehabilitation Involving Personal Tele-robotics—is based on designing a passive finger, thumb, and wrist orthosis for stroke rehabilitation (Amirabdollahian et al., 2014). SPO-F, the final design, is equipped with novel actuation mechanisms at the fingers and wrist and a motivational game environment based on ADL, combined with remotely monitored consistent interfaces (Ates et al., 2017). To make the devices inherently safe and integrable to home environment, a dynamic but passive mechanism is implemented, providing adaptable and compliant extension assistance. The device is targeted at patients who are able to generate some residual muscle control. Also, it utilizes physical interfaces developed by Saebo Inc. due to its proven track record in providing safe and comfortable interaction. An evaluation study on post-stroke patients using the device training with virtual reality games indicated the feasibility of home training using SPO-F, providing reports on the compliance and improvement of hand function after training (Nijenhuis et al., 2015) (Figure 1).

Liu et al. integrated a powered variable-stiffness elbow exoskeleton device with an sEMG-based real-time joint stiffness control to offer bilateral rehabilitation to patients suffering from hemiparesis (Liu et al., 2021). For a patient-specific approach ensuring human-like behavior patterns and facilitated coordinated movements, the device mirrors the dynamic movement captured from the unaffected side to generate stiffness-adapted motion to the contralateral side (Liu et al., 2018). The device also benefits from five passive DOFs for providing natural range of motion and minimizing misalignments between the robot and hand joints. Koh et al. introduced a soft robotic elbow sleeve for enabling flexion and extension of the elbow through passive and intent-based assisted movement execution. Further investigation is required to assess the efficiency of the device in neuro-muscular training (Koh et al., 2017).

Motivated by their prior research with HandSOME, Chen et al. attempted to target another population of patients with arm weakness instead of grasping impairment and developed a spring-operated wearable upper limb exoskeleton, called SpringWear, for potential at-home arm rehabilitation (Chen and Lum, 2018). With a total of five DOFs, SpringWear applies angle-dependent assistance to the forearm supination, elbow extension, and shoulder extension while incorporating passive joints for two other shoulder movements to allow complex and lifelike multi-joint movement patterns (Chen J. P. S. and Lum P. S., 2016). Over a ten-year iterative research cycle, Zhang et al. developed the wearable exoskeleton RUPERT—Robotic Upper Extremity Repetitive Trainer—for both clinical and in-home post-stroke upper-extremity therapy that incorporates five degrees of freedom of shoulder, humeral, elbow, forearm, and wrist (Zhang et al., 2011). Each DOF is supported by a compliant and safe pneumatic muscle (Huang et al., 2016). The device employs adaptive sensory feedback control algorithms with associated safety mechanisms. The developers claim that easiness in donning and operating the device and its graphic user interface excludes the need for the presence of a physical therapist.

As the end-effector of the human body, the hand takes the lead of ADL (Ates et al., 2017). Hence, improving the ability to perform ADLs is regarded as one of the main goals of physical/occupational therapy. To this end, Ambidexter, an end-effector type three DOF robotic device, has been developed for training hand opening/closing, forearm pronation/supination, and wrist flexion/extension (Wai et al., 2018). To meet the essential requirements for home systems, the cost was reduced while maintaining the effectiveness, the set-up process is easy and fast—taking less than one minute—and compactness and aesthetics were practiced carefully during the design process. A universal and ambidextrous grip is utilized to allow both left- and right-hand usage and to be adjustable to different hand sizes without changing the attachment. Both active and passive motion assistance in a game context is provided based on the ability of the user. Also, automatic customization is allowed by receiving user information from a mobile interfacethrough an integrated internet of things (IoT) network to monitor and communicate with the therapist. Ambidexter is lighter and smaller compared to its commercial counterparts, ReachMan and ReachMan2.

**FIGURE 2:**
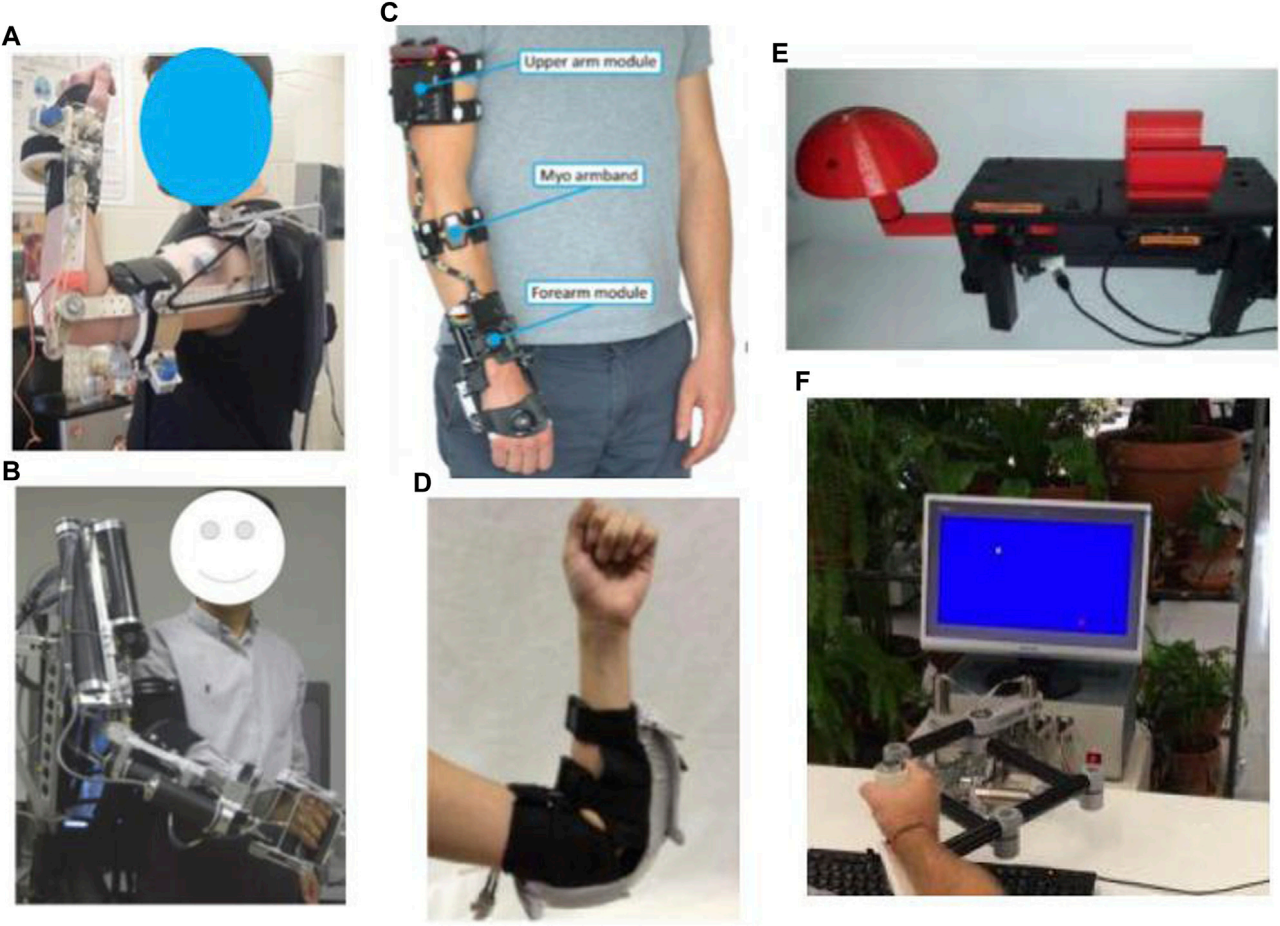
Robotic exoskeleton and end-effector devices for home-based upper-limb rehabilitation: **(A)** SpringWear (Chen and Lum, 2018), **(B)** RUPERT (Tu et al., 2017), **(C)** eWrist (Lambelet et al., 2020), **(D)** Soft robotic elbow sleeve (Koh et al., 2017), **(E)** Portable device for wrist rehabilitation (Ambar et al., 2017), and **(F)** HomeRehab (Díaz et al., 2018).

A novel home-based End-effector-based Cable-articulated Parallel Robot, PACER—Parallel articulated-cable exercise robot—, was developed by Alamdari et al. for post-stroke rehabilitation (Alamdari and Krovi, 2015). The device features a modular and reconfigurable design and is easy to assemble/disassemble. The device is able to assist and train the muscles involved in arm, forearm, and wrist motions. In 2018, Díaz et al. (2018) conducted research on converting a large pneumatic commercialized device, which had been designed for clinical therapy, into an electric and compact system for home rehabilitation by making it smaller, lighter, and cheaper, but maintaining the functionality (Díaz et al., 2018). It resulted in the development of HomeRehab, a desktop-type 2DOF robotic system intended to improve ROM and strength of the paretic hand in stroke patients. Designed for the home environment, all the components are concentrated in a small portable box, and the device can easily be placed on a home table. VR and gamification are also enabled through a standard PC communication and a novel low-cost force sensor. Force feedback allowed both assistive—the assist-as-needed approach—and resistive scenarios to be implemented during the therapy, depending on the therapist’s assigned task. Remote management is enabled by using wearable devices to record patient’s biosignals and providing a cloud-based communication system. A comparison between PupArm, a commercialized rehabilitation device for clinical settings, and HomeRehab indicated that, while offering similar outcomes, the latter is significantly lighter; 80 vs. 7 kg (Catalan et al., 2018) (Figure 2).

ArmAssist (Tecnalia R&I, Spain) is a portable, modular, easy-to-use, low-cost robotic system consisting of a tabletop module using omni-wheels, an arm, and hand gravity compensator orthosis aimed at post-stroke shoulder and elbow rehabilitation. Also, at the University of Idaho, add-on modules for wrist prono-supination and hand grasping training have been presented. Over the years, the device has been iteratively redesigned based on the updated requirements gained from clinical interviews, expert focus groups, and pilot tests with patients and therapists (Perry et al., 2012; Jung et al., 2013; Perry et al., 2016; Butler et al., 2017; Tomić et al., 2017).

As an intermediate step between high-power active and passive assistive robots, Westerveld et al. developed a low-power three-dimensional damper-driven end-point robotic manipulator, called active therapeutic device (ATD) (Westerveld et al., 2014). ATD provides a combination of passive arm weight support and assistance for functional reaching training. Increasing its potential for home-based therapy, the device deployed an inherently safe and compact system design. Washabaugh et al. designed a planar passive rehabilitation robot, PaRRo, that is fully passive yet provides multi-directional functional resistance training for the upper-limb (Washabaugh et al., 2019). This happens through integrating eddy current brakes with a portable mechanical layout that incorporates a large reachable workspace for a patient’s planar movements. The considered kinematic redundancies of the layout allow for posing direct resistance to the patients’ trajectories.

#### Commercially Available Devices

Based on the premise that the best way to reacquire the capability to perform a task is to practice that task repeatedly, Saebo Inc. proposed SaeboVR, a non-immersive VR rehabilitation system incorporating motivating games. These games are designed to simulate activities of daily living (ADL) and engage patient’s impaired arm in meaningful tasks aiming to evoke functional movements (Recover From Your Stroke With Saebo, 2017). The goal of the customizable tasks is to test and train user’s cognitive and motor skills such as endurance, speed, range of motion, coordination, timing, and cognitive demand, e.g., visual-spatial planning, attention, or memory, under the supervision of a medical professional in a home setting. The device includes a Provider Dashboard application that enables the medical professional to view patient performance metrics and participation history while providing audiovisual feedback and graphic movement representation for patients.

To target a specific group of patients with various treatment options, this device can be upgraded with additional technologies, e.g., SaeboMas, SaeboRejoyce, or SaeboGlove, which can be integrated into the virtual environment. SaeboGlove, a functional hand orthosis, combined with electrical stimulation, has been shown to be beneficial for functional use of moderately to severely impaired hands in sub-acute stroke patients (Franck et al., 2019). SaeboMAS, a zero-gravity upper extremity dynamic mobile arm support device, provides the necessary weight support in a customizable manner, facilitating exercise drills and functional tasks for the individuals who have arm weaknesses (Runnalls et al., 2019). The usage of this technology will potentially allow patients with proximal weakness to reach a larger anterior workspace and engage in more versatile functional tasks and exercises that would have otherwise been difficult or impossible. The SaeboReJoyce is an upper extremity rehabilitation computerized workstation that includes pre-installed neurogaming software and offers task-oriented and customizable games. The workstation is composed of two components. A lightweight height-adjustable and portable gross motor component makes the device useful for sitting, standing, and lying positions and enables the execution of exercises and tasks in all directions and planes. A fine motor component aims at improving necessary dexterity for daily tasks by incorporating various grip and pinch patterns, such as spherical grasp, grip strength, wrist flexion/extension, tip to tip pinch, and pronation/supination, among others. Several studies have been conducted to prove the efficacy and results of Saebo products (Adams et al., 2018; Doucet and Mettler, 2018; Runnalls et al., 2019).

Another home rehabilitation device for post-stroke patients is WeReha, which is intended to be used for hand impairments with the possible remote supervision of physiotherapists (Bellomo et al., 2020). The primary purpose of this device’s invention is to train the fine movements of the hand and simultaneously stimulate the cognitive aspect, exploiting the biofeedback technology. It can track patient movements with the 3D printed “smart” objects equipped with inertial sensors while transmitting data via Bluetooth to the software. The software processes the data and produces visual-auditory feedback to guide the patient. Such guidance enables the patients to correctly execute various motor tasks through the exercises in the specially designed and studied video games that allow them to exercise the grip of finer or larger objects. Moreover, simple and effective gamification is based on indexes of rotation, flexion-extension, and prono-supination of the upper-limb.

The soft extra muscle (SEM) Glove by Bioservo Technologies AB, Sweden (BIOSERVO, n.d.), was developed to improve the hand’s grasping capacity by providing additional finger flexion strength. This novel technical solution simultaneously mimics a biological solution and functions in symbiosis with the biological system that excludes the need for an external mechanical structure to achieve controlling and strengthening effects. The servo device uses artificial tendons connected to electrical motors that actuate finger movements by creating pulling forces. The device features intention detection to apply proportional finger flexion strength facilitating grip or object manipulation, benefitting from control algorithms that are based on tactile sensor signals located on the tip of fingers. The efficacy of SEM Glove has been evaluated for improving gross and fine hand motor functions for at-home rehabilitation for people with impaired hand function after high-level spinal cord injury, yet further investigation is required for its efficiency for post-stroke rehabilitation (Nilsson et al., 2012; Osuagwu et al., 2020). Based on the SEM Glove technology and with the aim of extending its application for stroke rehabilitation at home, Radder et al. designed IronHand, a lightweight and easy-to-use soft robotic glove that thanks to the soft and flexible materials used for its fabrication, accommodates wearable applications (Radder et al., 2019). The IronHand, formerly known as HandinMind (HiM) project, offers an easy-to-use combination of assistive functionality during ADL and therapeutic functionality through a training context within a motivating game-like environment (Prange-Lasonder et al., 2017). They allow individuals with reduced hand function to use their hand(s) during a large variety of functional activities. The therapeutic functionality of the device incorporates a therapeutic software platform for patient assessment and database and it covers therapy goals of simultaneous finger coordination, hand strength and sequential finger coordination (Radder et al., 2018). The assistive functionality provides extra strength to the grip of fingers after the active contribution of the user’s grip force, and an intention detection logic ensures that extra force proportional to that of the user is activated. IronHand is one of the first user trials that applied and tested a fully wearable robotic system in an unsupervised home setting to support hand function during an extended period of multiple weeks. Findings from this extensive trial indicated improvements in unsupported handgrip strength and pinch strength (Radder et al., 2019).

In an attempt to transfer Gloreha—Hand Rehabilitation Glove—Professional, a wearable hand rehabilitation hospital device, to a home setting, Gloreha Lite has been miniaturized and specifically designed for home use in a safe and feasible way for hand rehabilitation (Bernocchi et al., 2018). Gloreha Lite is a portable, lightweight, and space-saving glove-brace (Aggogeri et al., 2019). The robotic glove represents a relevant adjunct intervention to intensify activity-based therapy, integrating the principle of neuroplasticity with the intensity of treatment (Proulx et al., 2020). It provides computer-controlled passive mobilization of the fingers. Before each exercise drill, a 3D-simulated preview of the movement is presented on the monitor, and during the performance of the movement, a simultaneous 3D simulation of the movement is displayed as it is being performed. Bernocchi et al. evaluated and demonstrated the feasibility and safety of the device for in-home therapy and indicated improvement of functional capacity of the paretic hand. They also demonstrated that the acquired benefits on strength and dexterity were maintained over time. The majority of patients completed the entire course of the program while performing all the prescribed home exercises.

**FIGURE 3:**
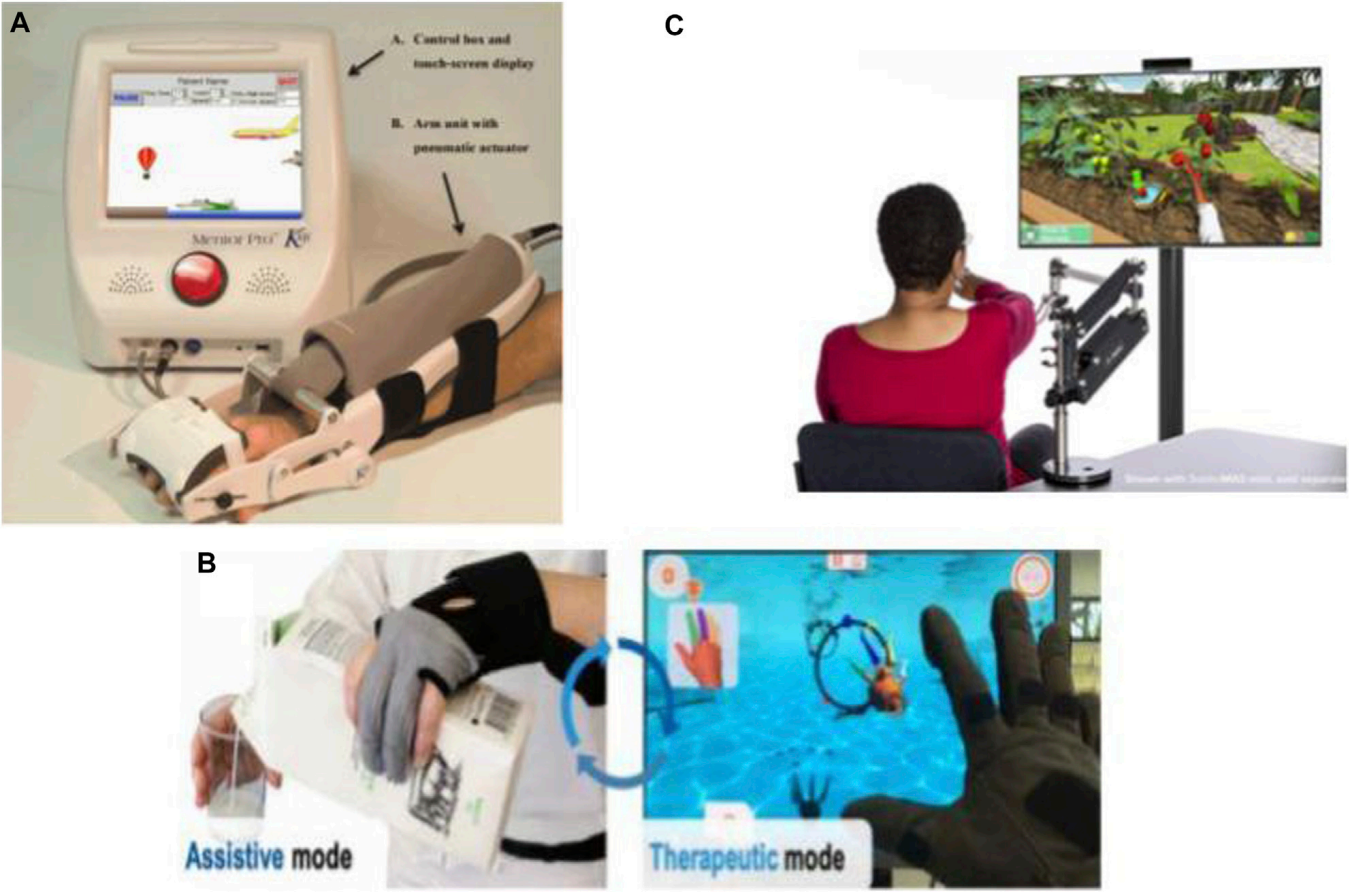
Commercialized robotic devices for home-based upper-limb rehabilitation: **(A)** The Motus Hand (Butler et al., 2014), **(B)** IronHand (Radder et al., 2019), and **(C)** SaeboVR (Recover From Your Stroke With Saebo 2017).

The Motus Hand (Motus Nova, n.d.) (https://motusnova.com/hand), previously known as Hand Mentor Pro, is a portable robotic device designed to enhance active flexion and extension movements of wrist and fingers along with motor control of the distal upper limb. The device deploys pneumatic artificial muscles for simulating dorsal muscle contraction and relaxation. The Motus Hand has been classified as an FDA class 1 device presenting non-significant risk (NSR). Several clinical trials investigated and supported the clinical efficiency, feasibility, and user-friendliness of the Motus Hand for in-home telerehabilitation among subacute to chronic post-stroke (Linder et al., 2013a; Linder et al., 2013b; Butler et al., 2014; Wolf et al., 2015) (Figure 3).

Hyakutake et al. investigated the efficiency and feasibility of home-based rehabilitation involving the single-joint hybrid assistive limb (HAL-SJ) (Hyakutake et al., 2019). Drawing on the “interactive biofeedback” theory, HAL-SJ is a lightweight power-assisted exoskeleton on the elbow joint triggered by biofeedback for assisting the patient in the voluntary movements of the affected upper limb.

### Lower-Limb

#### State-of-the-Art: Systems in the Literature

Many developed lower-limb robotic systems offer rehabilitation in sitting/lying positions for stroke patients who cannot stand or walk safely (Eiammanussakul and Sangveraphunsiri, 2018). In this approach, the patients may exercise more independently with no safety concerns like falling. Therefore, compared to the other lower-limb rehabilitation principles, such as treadmill gait trainers, this kind of lower-limb device shows considerable potential for in-home therapy. Moreover, these robots are potentially suitable for home environments, as they can be smaller, lighter, and portable.

Spasticity of the limbs is one of the most common impairments ensuing onset of stroke. It puts patients at a high risk of developing foot deformity. Hence, treating the spasticity of lower extremities to prevent any deformity and facilitate ankle muscle activities during the acute phase and even after the long bedridden period is of utmost importance. In order to reduce or prevent the occurrence of spasticity at later stages, Low et al. developed a soft robotic sock, which can provide compliant actuation to simulate natural ankle movements in the early stage of stroke recovery. The soft robotic sock controls the internal pneumatic pressure of the soft extension actuators to assist the patient in ankle dorsiflexion and plantarflexion (Low et al., 2018; Low et al., 2019). For the same purpose, Ren et al. also developed a wearable ankle rehabilitation robotic device capable of delivering in-bed stroke rehabilitation in three training modes, active assistive training, resistance training, and passive stretching (Ren et al., 2017). These aforementioned devices mainly target people with acute stroke; Nonetheless, to treat chronic stroke survivors who have already developed ankle-foot deformities or imbalanced ankle muscles, Lee et al. developed a relatively small, lightweight, and user-friendly rehabilitative system, called Motorized Ankle Stretcher (MAS) (Beom-Chan et al., 2017; Yoo et al., 2019). The MAS consists of two linear in-line actuators, each connected to a platform for generating ankle dorsiflexion and eversion. The system requires patients to perform exercises in the standing position, with a walker positioned in front of them for safety concerns. The above-mentioned devices have not yet been implemented in home-setting but are deemed potentially feasible by the authors in terms of safety, size, portability, complexity, and user-friendliness. However, the clinical efficiency of these devices for in-home treatment requires further investigation.

As one of the first home-based EMG-controlled systems, Lyu et al. (2019) developed a knee exoskeleton within a game context to be used while the subject was seated on the chair wearing the exoskeleton. Utilizing four active DOF at both hip and knee, the exoskeleton improves knee joint movement stability and accuracy by strengthening anti-gravity knee extensor muscles. The robot can be generally considered a successful effort at designing a home-based system by being adjustable to the wearer’s leg length, with a quick setup time of approximately 1 min, and considering safety cautions in all stages of software, electrical, and mechanical. Initial testing on healthy subjects represented promising results on the possibility of carrying out early rehabilitation by this device, the amount of muscle activation by the participants, and the timing of that activation.

**FIGURE 4:**
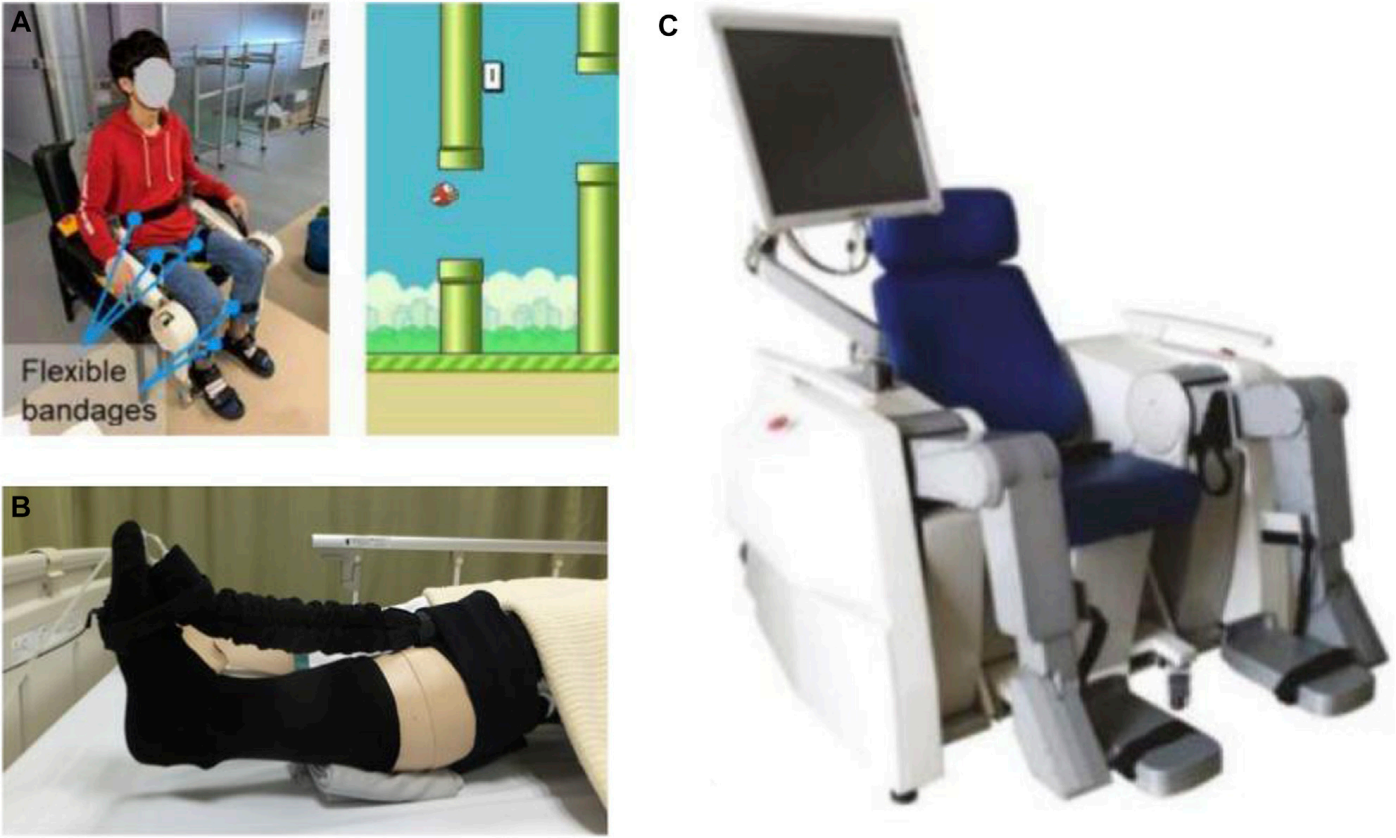
Robotic devices for sitting/lying home-based lower-limb rehabilitation: **(A)** EMG-Controlled Knee Exoskeleton (Lyu et al., 2019), **(B)** Soft Robotic Sock (Low et al., 2019), and **(C)** LLR-Ro (Feng et al., 2017).

Another example of lower-limb devices is a multi-posture electric wheelchair developed by Chen et al. with a lower-limb training function. Combined with virtual reality games and a tele-doctor–patient interaction, this device forms an intelligent rehabilitation system suitable for home therapy (Chen S. et al., 2017). Apart from rehabilitation training, it can be used as an everyday wheelchair. This hybrid nature of the design has made it economically efficient. The wheelchair is equipped with four linear motors to carry out the training function and the lying/standing process, and is controlled by a cell phone interface. The system also benefits from a communication platform through web-based interfaces for patients and doctors. As another genre of lower-limb rehabilitation robots seemingly viable for home implementation, a sitting/lying Lower Limb Rehabilitation Robot (LLR-Ro) was developed containing a moveable seat and bilaterally symmetrical right and left leg mechanism modules, each comprising the hip, knee, and ankle joints. This device benefits from mechanical, electrical, and software safety features and an amendment impedance control strategy to realize good compliance (Feng et al., 2017) (Figure 4).

One of the most prevalent lower-limb impairments following hemiplegia in post-stroke patients is asymmetric gait patterns and balance dysfunction. These impairments can adversely affect the quality of life as they lead to compensatory movement patterns, slowed gait speed, limited functional mobility, which results in reduced performance of the activities of daily living and increased risk of experiencing falls. Therefore, regaining autonomous gait and improving independent walking ability should be among the top priorities of rehabilitation interventions post-stroke. Aimed at improving stability and walking capacity, Morone et al. introduced i-Walker, a robotic walker for overground training with embedded intelligence that provides asymmetrical assistance as needed by detecting the imposed force by the user to adjust the amount of help to the impaired side (Morone et al., 2016).

Developing wearable robots for lower-limb treatments has increasingly gained traction for their capability to facilitate ambulatory rehabilitation delivery. Among them, Gait Exercise Assist Robot (GEAR), proposed by Hirano et al. in collaboration with Toyota Motor Corporation, is a wearable knee-ankle-foot robot (only for the paralyzed leg) integrated with a low floor treadmill and a safety suspending device (Hirano et al., 2017; Tomida et al., 2019). The Lokomat (Hocoma AG, Volketswil, Switzerland) is a commercial widely used exoskeleton-type robot for gait training worn over both lower extremities, consisting of a combination of adjustable orthoses, a dynamic bodyweight support system, and virtual reality for providing sensory-motor stimulation (van Kammen et al., 2017). Although the clinical efficacy of wearable devices for lower extremity is supported by a growing body of evidence (Hidler et al., 2009; Tomida et al., 2019; Mehrholz et al., 2020), only a number of them are realizable in a home setting with modifications addressing various factors, e.g., safety issues, size, weight, portability, complexity, and cost. Among robotic rehabilitation devices of the past ten years, those provisioned for home therapy only requiring further investigation validating their clinical efficiency at home are presented.

Walking Assist for Hemiplegia (WA-H) is a portable, lightweight, modular, and wearable exoskeletal robot supporting the hip and knee joint movements that, by providing customized gait training, can be used in various environments depending on the degree of impairment in patients. WA-H has an inherently safe design in which all robot joints mechanically limit the movements occurring beyond the natural range of motion. The device features a passive joint simulating the weight shift occurring during walking in the hip joint in the coronal plane (Moon et al., 2017; Sung et al., 2017).

**FIGURE 5:**
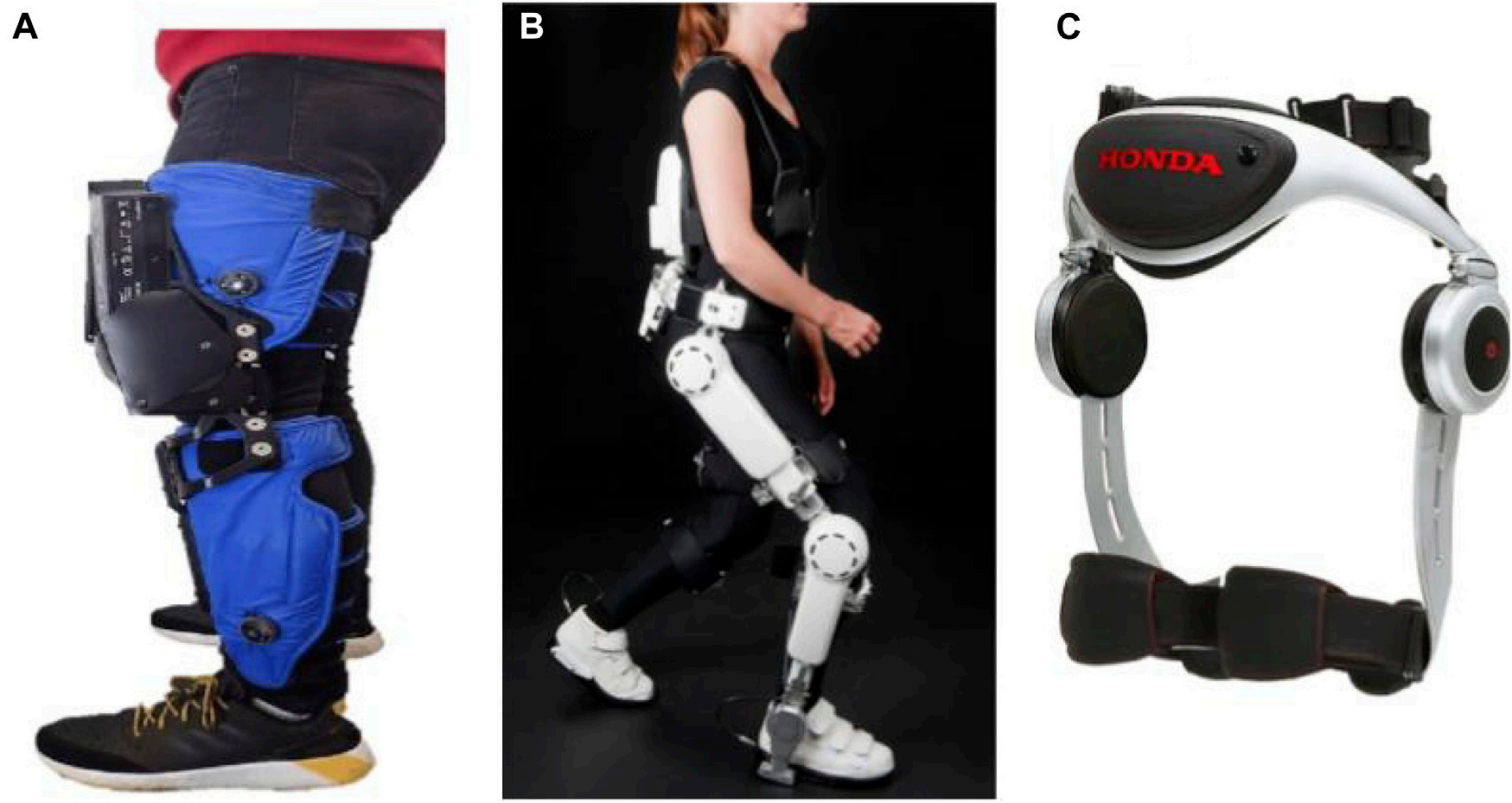
Robotic devices for in-home walking training: **(A)** AlterG Bionic Leg (Wright et al., 2020), **(B)** HAL lower-limb exoskeleton (Anneli Nilsson et al., 2014), and **(C)** SMA (Buesing et al., 2015).

As both rehabilitation and welfare robot, Curara^®^ is a wear robot that assists hip and knee joints in both impaired and unaffected legs simultaneously with no rigid connection between joint frames, resulting in a higher degree of freedom. Prioritizing user-friendliness in terms of ease in don/doffing and minimizing the restraining stress against the natural human movement, Mizukami et al. adopted a non-exoskeletal structure coupled with a synchronization-based control system, introducing the ability to feel what natural movement would be like. Due to the absence of any rigid connection between joint frames, the device provides a high degree of freedom for patient movement (Tsukahara and Hashimoto, 2016; Tsukahara et al., 2017; Mizukami et al., 2018). Lee et al. developed a smart wearable hip-assist robot for restoring the locomotor function, the Gait Enhancing and Motivating System (GEMS, Samsung Advanced Institute of Technology, Suwon, South Korea). GEMS is equipped with an assist-as-needed algorithm for delivering active-assistance in hip extension and flexion (Lee et al., 2019; Lee et al., 2020) (Figure 5).

Interposed between active and passive training robots, Washabaugh et al. proposed their eddy current braking device on a knee brace as a wearable passive alternative that provides functional resisted gait training while adhering to features required for home-based devices (Washabaugh et al., 2016; Washabaugh and Krishnan, 2018).

#### Commercially Available Devices

There are several commercially available lower-extremity rehabilitation robots, and those exhibiting potential for home therapy are presented. The wear overground robotic Stride Management Assist (SMA^®^) is developed and available for purchase (Honda Global, 2020) by Honda that assists hip joint movements for increasing walking performance independence (Buesing et al., 2015). The ReWalk ReStore™ is a soft exosuit designed and introduced by ReWalk (2019) for actively assisting paretic ankle plantarflexion and dorsiflexion for the propulsion and ground clearance walking subtasks (Awad et al., 2020). ReWalk Robotics also proposed the ReWalk Personal 6.0 System as a customizable exoskeleton with motors at the hip and knee joints specifically designed for all day home-use (ReWalk, 2015). EksoNR is an FDA-cleared ambulatory exoskeleton featuring adaptive gait training and posture support, yet it is currently being tested in a clinical trial with stroke patients for evaluating its clinical efficiency (Carlan and singleorigin, 2020) (https://eksobionics.com/eksonr/).

Different exoskeletons have been developed based on the HAL’s technology to offer active motion support systems with a hybrid control algorithm, Cybernic Voluntary Control for providing physical support associated with the patients’ voluntary muscles activity and Cybernic Autonomous Control that utilizes characterized movements of healthy subjects and adopts the motion patterns in accordance. One such device has been described in the prior section for elbow rehabilitation. Kawamoto et al. also, based on this technology, developed the single-leg version of the HAL, an exoskeleton-based robotic suit for independent supporting of the ankle, knee and hip joints (Kawamoto et al., 2009). Kawamoto et al. and then Nilsson et al. investigated the efficiency of this exoskeleton for intensive gait training for chronic and acute, respectively, hemiparetic patients (Kawamoto et al., 2013; Nilsson et al., 2014). The device is commercially available in Japan (Cyberoyne, 2021).

The AlterG Bionic Leg (AlterG, 2015; AlterG, n.d), a portable dynamic battery-operated over-ground wearable lower extremity orthosis, provides adjustable progressive mobility training by supplementing existing muscle strength, providing audio-sensory feedback and mobility assistance during rehabilitation (Stein et al., 2014; Iida et al., 2017; Wright et al., 2020). This dynamic orthosis supports knee mobility when standing or walking by providing external support to the lower extremity and assisting the patient in aid of weight shifts and knee movement and can be worn in a manner similar to an orthopedic knee brace.

## Design Paradigm

The interdisciplinary field of rehabilitation requires the simultaneous employment of a range of expertise, including engineering, medicine, occupational therapy, and neuroscience, especially due to the lack of enriched research in motor learning principles for optimized post-stroke motor recovery (Krakauer, 2006; Brewer et al., 2007). A successful home rehabilitation device can be designed within a certain sequence of steps that incorporate all of the aforementioned expertise, as skipping one could prevent achieving optimum outcomes.

Mechatronic home-based systems for post-stroke therapy are based on four basic components: 1) a mechatronic device delivering rehabilitation intervention, 2) a control system ensuring proper performance of the system, 3) interactive interfaces for patients and medical professionals who provide remotely supervised therapy, 4) a communication system gluing the whole system together.

To provide a classified roadmap for assisting researchers who aim at further developing this field, a design paradigm is proposed to form a guideline on developing each component based on the engineering design process.

### Post-Stroke Rehabilitation and Treatment Interventions

Among different post-stroke symptoms, motor deficits are the most commonly recognized impairments that affect the face, arm, and leg motor functions. These impairments result in various manifestations, including impaired motor control, muscle weakness or contracture, changes in muscle tone, joint laxity, spasticity, increased reflexes, loss of coordination, and apraxia (Basteris et al., 2014; Hatem et al., 2016). To recover lost function and aid motor recovery, many rehabilitation interventions have been developed based on neurorehabilitation principles (Basteris et al., 2014). So as to develop a robot-assisted therapy that offers maximal motor function recovery, it is essential to employ interdisciplinary research on the broad spectrum of post-stroke disabilities and their corresponding rehabilitation protocols. The outcomes could help create a clear picture of the target users, establish target and rehabilitation program specifications, and derive requirements from the needs of all stakeholders, the largest being the therapists and the patients. Note that it is essential for the device to be accepted by medical professionals. From the therapists’ point of view, the crucial features for iterative design and modification of each rehabilitation robot must be surveyed and practiced carefully.

Engineers should develop the rehabilitation system based on multiple contributing factors, including the part of the limb being trained, the targeted stage of recovery, the severity of initial motor deficit, range of movements in the paretic limb, grade of spasticity, age, and individual patient’s characteristics. Depending on the patient, it is known that motor impairments can induce disabilities in several functions, such as range of motion, speed, coordination, cadence (steps/minute), balance, precision, the ability to regulate forces, muscle strength, and energy efficiency (Perry et al., 2011). Physiological measurements during rehabilitation, i.e., heart rate, blood pressure, body temperature, etc., assist in sensing the patient’s status during therapy and their capability to do exercise, and in turn, offer a foundation for determining the dosage of assigned tasks based on one’s capability (Solanki et al., 2020). Monitoring physiological parameters could also be utilized for detecting the user’s psychological state, in terms of mood, motivation, engagement, etc., and lead to modification of the course of therapy accordingly (Novak et al., 2010). It is important to tailor treatment strategies to the goals of improving one or a combination of these functional disabilities. Current robotic rehabilitation systems incorporate a variety of neurorehabilitation strategies. These strategies include constraint-induced movement therapy (CIMT), repetitive movement training, impairment-oriented training, explicit learning paradigms such as bilateral training, implicit training, and functional task paradigms (Brewer et al., 2007). Given training goals as guidelines, a combination of these interventions can be utilized in order to develop a reasonable user-specific program with respect to the aforementioned critical factors (Hatem et al., 2016), as different types of tasks are necessary to retrain all lost function.

Recovery would benefit if scientific principles behind post-stroke motor learning were incorporated into the design of the rehabilitation device. Under the assumption that performance improvement is dependent on the amount of practice, most current mechatronic devices for post-stroke therapy are solely based on the repetition of a single task, termed “massed practice” (Brewer et al., 2007). It is important to note that, although repetition is the key to improving within-session performance, other critical factors must be considered while scheduling rehabilitation protocols. As Krakour et al. state, there are two crucial questions to ask before developing any rehabilitation system: “whether gains persist for a significant period after training and whether they generalize to untrained tasks” (Krakauer, 2006; Kitago and Krakauer, 2013). There is evidence that “distributed practice,” which means inserting more extended rest periods between repetitions, e.g., increasing the number of sessions while decreasing session duration, promotes retention (Krakauer, 2006; Kitago and Krakauer, 2013). While limitations of clinical therapy preclude the proper implementation of this method, home-based therapy provides the opportunity of distributed task scheduling in a way that it can always be at the patients’ disposal.

For rehabilitation interventions to be meaningful, learned tasks must generalize to new tasks or contexts, especially real-world tasks. Introducing variability to training sessions, though worsening the patients’ performance in the short term, improves their performance in retention sessions and also increases generalization by representing each task as a problem to be solved rather than just memorized and repeated (Krakauer, 2006; Kitago and Krakauer, 2013). Contextual interference is a concept used to introduce variability to the task by random ordering between several existing tasks. Moreover, recovery of function to increase patient autonomy is another important aspect of rehabilitation. It seeks to consider training for true recovery, as well as, compensatory mechanisms—respectively accomplishing task goals by recruiting the affected muscles or alternative muscles (Krakauer, 2006; Kitago and Krakauer, 2013). Nevertheless, when establishing goals for rehabilitation interventions, there has to be a clear distinction in mind between these two, true or compensatory recovery, as they may make differential contributions to the treatment plan.

### Mechatronic System

Once the target group and treatment plan have been identified, the design criteria, including requirements and constraints, need to be established and prioritized to fit the need. Since the device is being designed for the home setting, certain factors are introduced, and some others become more prominent—safety, adaptability to the home setting, the autonomy of patients, aesthetic appeal, affordability, to name a few (Carbone et al., 2018; Chen et al., 2019).

#### Design Criteria for Home-Use

In addition to the general criteria, the adoption of each home rehabilitation solution requests specific features of the device itself. For example, in the case of exoskeletons, besides absolute safety when worn, lightness, wearing ease, comfortability, and smoothness, there should be an absence of friction and allergenic factors as it is in contact with the skin. So the device should guarantee a high tunability and reliability (Borboni et al., 2016). Considering all of the factors and criteria, a solution has to be adopted, which offers a satisfactory compromise to each of the existing issues and requests of all parties involved, as all of the requirements affect the device’s structure. Also, there are some optional and preferable criteria, among which expandability and upgradability are favorable to cover a wide range of disabilities and possible treatment methods. To this end, modularization benefits both manufacturers and customers for it increases diversity and a variety of available options and enables interchangeability and compatibility. This way, various gadgets could be developed to be integrated into a wide range of existing home-based devices, which in turn enhances both acceptability and functionality. For example, Amirabdollahian et al. in the SCRIPT project and Kutlu et al. in their home-based FES rehabilitation system utilized commercialized Saebo module, SaeboMAS arm support (Amirabdollahian et al., 2014; Kutlu et al., 2017). Moreover, the donning aspect of robotic devices in an unsupervised context should be underlined. Therefore, attachment mechanisms should be designed to enable facilitated and independent donning and doffing of the device by the patient. In this regard, Lambelet et al. designed the eWrist to offer easy and fast donning/doffing to enable single-handed mounting of the device for hemiparetic patients, significantly promoting the autonomy of the patient were they to train independently (Lambelet et al., 2020). Equivalently, Fischer et al. realized facilitated donning of X-Glove by means of adopting a two-separate component design, a zipper on the palmar side, and a flexed wrist posture (Fischer et al., 2016). It should be flexible to be used in different positions, such as sitting/lying for bedridden or chaired individuals, and also light enough to be easily transportable. Another criterion that needs to be taken into account is that the device’s functioning noise must be as low as possible to be acceptable by the patient (Borboni et al., 2016).

#### Mechanism Type

Having design criteria and target functionality in mind, the designer has to decide the mechanism type. The type of the mechanism and the treatment options are correlated; for example, additional movement protocols can be utilized based on the number of arms. In general, human limb rehabilitation robots are divided into two groups based on the target limbs: upper-limb rehabilitation devices and lower-limb rehabilitation devices, each divided into several subgroups. Based on motion systems, upper-limbs are categorized into exoskeletons or end-effector devices. Based on the patient’s posture, lower-limb devices can be designed to be used in sitting/lying positions or standing positions with the help of body and robot weight support.

In his study in 2019, Aggogeri et al. categorize robotic rehabilitation technologies into end-effector or exoskeleton devices based on design concepts (Aggogeri et al., 2019). End-effector devices, also known as endpoint control, determine the joint level movements by recreating dynamic environments corresponding to ADL. End-effector devices may be dedicated to hand rehabilitation or integrated into more complex structures for arm recovery. Attached to the user’s limbs, exoskeletons are wearable robots aiming at enhancing their movements. Focusing on the patient’s anatomy, each degree of freedom of the device is aligned with the corresponding human joint. Exoskeletons should be compliant with the user’s movements and deliver part of the power required by the movements. Therefore, the mechanical axes of exoskeleton joints and anatomical joints should be aligned to prevent patient discomfort and not obstruct natural limb movement (Fischer et al., 2016). In designing exoskeletons, the high sensitivity of stroke survivors to the applied mechanical load on their paretic limb makes the weight of the device an important factor to be considered. Reducing the applied load on the impaired limb can be achieved through locating the components not directly involved in actuation—e.g., battery and controller—on more proximal rather than distal body parts. For example, Fischer et al. provided an upper arm module for locating the battery and electronics, resulting in the reduced weight of the forearm module located on the distal part of the arm (Fischer et al., 2016). Equivalently, in the ReWalk Restore lower-limb exosuit, the actuation pack is worn at the waist so that the larger proportion of device weight is located proximally (Awad et al., 2020).

Comparing these two different approaches, end-effector robots are more flexible than exoskeleton devices in fitting the different sizes, require less setup time, and increase the usability for new patients. Besides, end-effector mechanisms are also generally ambidextrous. On the contrary, exoskeletons should be fully user-adjustable and therefore require more complex control systems. While both distal and proximal joints are constrained in exoskeleton devices, end-effector robots merely constrain the distal joints (Aubin et al., 2013). Therefore, explicit control of each individual joint is only possible with exoskeleton devices. The limited control of end-effector robots could result in abnormal movement patterns in patients. In contrast, due to the direct controllability of individual joints in exoskeletons, these abnormal postures or movements are minimized (Aggogeri et al., 2019).

In conventional exoskeleton mechanisms, the rigidity of the frames and fixed straps poses an issue on their wearability and usability. The heaviness and bulkiness of such frames result in high energy cost and also affects the natural gait dynamic and kinematics of the patient. Hence, soft orthotic systems have been developed as an alternative to traditional rigid exoskeletons (Lee et al., 2019). In this regard, soft robots have shown promising potential to be adopted for at-home rehabilitation. In their study, Polygerinos et al. argue that soft wearables could further advance home-based rehabilitation in that they provide safer human-robot interaction due to the use of soft and compliant materials, a larger range of motion and degrees of freedom, and increased portability. The materials used for the fabrication of these robots are inexpensive, making these devices affordable. Also, soft material makes these devices inherently lighter and, therefore, more suitable for rehabilitation purposes. Another advantage of soft robotic devices over conventional rehabilitation robots is that they can be fully adapted to the patient’s anatomy offering a more customizable actuation (Polygerinos et al., 2015).

In rehabilitation devices, it is essential to improve physical human-robot interaction (pHRI). For each type of rehabilitation device, the recruitment of different engineering methods is required for such improvements. This interaction is fundamentally affected by the mechanisms that should be designed by taking sophisticated biological features and activities into account. By considering the compliance/stiffness factor, modes of actuation and transmission need to be selected in a systematic way. Control methods also affect pHRI (Gull et al., 2020).

#### Degrees of Freedom

The number of active and passive DOFs determines the system’s functionality. They condition the workspace in which the joints are capable of moving, indicating the assistance/rehabilitation, which is needed to be delivered to each joint (Shen et al., 2020). Patients’ anatomy should be incorporated into the design when determining a reachable workspace based on anthropometric norms of the end-user (Washabaugh et al., 2019). By reviews on upper and lower-limb devices, it can be figured out that the majority of the developed devices profit from certain degrees of freedom that are compatible with the human body’s anatomy. Anatomically speaking, in the upper extremity, often simplified to have seven degrees of freedom, the shoulder is simplified as three rotary joints achieving extension/flexion, adduction/abduction, and internal/external rotation. Elbow and forearm are simplified to provide extension/flexion and pronation/supination movements, respectively. Lastly, the wrist achieves extension/flexion and radial/ulnar deviations. The seventh DOF in the upper extremity’s joint space poses challenging complications since the maximum DOFs in the task space is six. Not only does this require us to have a firm understanding of how a human resolves this redundancy issue, but also such understanding must be taken into consideration when designing rehabilitation device mechanisms (Shen et al., 2020). Furthermore, fingers are simplified as joints capable of achieving flexion/extension and abduction/adduction movements.

In the lower extremity, the hip is simplified as three rotary joints to achieve flexion/extension, abduction/extension, and internal/external rotation. The knee achieves pure sagittal rotation and flexion/extension. And finally, the ankle, simplified into three rotation joints, achieves plantar/dorsiflexion, eversion/inversion, and internal/external rotation (Shi et al., 2019). The kinematic models should be developed by considering the anthropometric and morphology of human body structure in accordance with the command-and-control possibilities of actuated joints (Dumitru et al., 2018; Cardona and Garcia Cena, 2019). Joint movements during upper and lower-limb movements cause misalignments between the exoskeletal and human joint axes that need to be adjusted and fit to the position of joints. Some exoskeletal devices have addressed this issue by providing passive joints to trace the joint movements to enable of the wearer’s natural range of motion. For example, Sung et al. equipped WA-H with passive hip joints in the coronal plane to enable weight shifting during walking (Sung et al., 2017). Also, Liu et al. incorporated three passive shoulder joints in their design to minimize the misalignments (Liu et al., 2021).

#### Modeling Tools

Modeling tools are of paramount importance in the design of rehabilitation devices for both robot and musculoskeletal modeling. Rigid body simulation programs or general-purpose simulation software such as Adams, Matlab, and Modelica can be used to evaluate the mechanics and control aspects. Also, computer-aided design software, such as CATIA and SOLIDWORKS, could be used to design, simulate, and analyze these robotic mechanisms. To simulate and control soft robots, SOFA, an open-source framework, can be used. It provides an interactive simulation of the mechanical behavior of the robot and its interactive control. It is also possible to model a robot’s environment to be able to simulate their mechanical interaction.

On the other hand, the heavy dependence of design parameters upon the targeted application requires careful analysis of the human body anatomy to design the device by considering the end-user application (Gull et al., 2020). In turn, programs such as OpenSim and AnyBody can evaluate and predict the effect of the device on the human musculoskeletal system for any given motion. For example, in order to generate a digital exoskeleton model, Bai et al. exported the designed exoskeleton in CAD, SolidWorks, to AnyBody (Bai and Rasmussen, 2011). When it comes to musculoskeletal systems, software tools can significantly facilitate the process of derivation of the motion equations to model muscle force and path. Various musculoskeletal software packages are commercially available such as SIMM, OpenSim, AnyBody, and MSMS. Among these software packages, OpenSim is free and open-source software, and MSMS is a free software (Cardona and Garcia Cena, 2019). Muscle activation and muscular contraction dynamics of musculoskeletal models can also be used as a reference input signal for real-time controlling methods. For example, Liu et al. utilized an s-EMG-driven musculoskeletal model to adjust the stiffness control based on the patient’s physical status and assigned task requirements (Liu et al., 2018).

Two commonly used dynamic modeling methods are Newton-Euler and Lagrange’s methods. In the Newton Euler method, by solving the Newton-Euler equation, the robot’s internal and external forces are extracted. In Lagrange’s method, which is based on the system’s energy, the external driving force/torque of the system can be calculated. In 2019 Zhang et al. drew a comparison between these two methods and provided a table to demonstrate the differences between these methods. Derivation analysis in the Newton-Euler method is more complicated than the Lagrange method, but calculations in the Newton-Euler method are large and heavier to compile, while in the Lagrange method, are easily compiled. In the Newton-Euler method, in addition to the driving force/moment, internal forces can be obtained (Zhang et al., 2019). Dynamic simulation can be carried out in Adams environment while theoretical calculations are performed in MATLAB. Zhang et al. use these simulations to provide a basis for the optimal design of the structure and the selection of the motor (Zhang et al., 2019).

#### Actuation and Transmission

The design of the device, further completed and integrated with the detailed design of chosen actuators, the transmission system, and the sensing system, should be presented at the next step. As there are myriad options to choose from, the designer has to weigh his/her options against the design criteria and their allocated importance and priority.

Efficient actuator design is important for home-based rehabilitation systems since these systems should be compact. Therefore, as the main powering elements, small-sized actuators that have a high power-to-weight ratio are required as they are capable of producing high torques with precise movement (Gull et al., 2020). Actuators should be chosen based on the target application. Three main categories of actuators are electric motors, hydraulic/pneumatic actuators, and linear actuators.

Electric motors are used for their quick responses and capability of providing high controllability and controlled precision. However, they have a low power-to-mass ratio and are usually expensive. Pneumatic actuators can yield high torques but could save self-weight. Nevertheless, by using these types of actuators, the portability of the system is compromised due to the accompanying inherent components such as pump, regulators, valves, and reservoirs. Another factor that makes these types of actuators unsuitable for home-based systems is that they require maintenance since lubricant/oil leakages could be problematic for users. In hydraulic and pneumatic actuators, control is less precise, and hence the safety cannot be ensured. According to Shen et al. and Gul et al., these actuators are not suitable for providing assistance or therapy or for rehabilitation purposes due to their possessing high impedances; however, some studies utilize them because of their ability to provide high power. Ultimately, based on the studies done by Gull et al., Series Elastic Actuators (SEAs) by reducing inertia and user interface offer a safe pHRI and can achieve stable force control (Gull et al., 2020). Tuning the stiffness of the transmission system is one of the approaches to achieve a specific level of compliance. In this regard, Jamwal et al. suggest using variable/adjustable stiffness actuators for rehabilitation purposes since these actuators offer safer human-robot interaction due to their ability to minimize large forces caused by shocks (Jamwal et al., 2015). Especially, employing SEA in lower-limb robots offers the advantage of a facilitated control-based disturbance rejection by improving tolerance to mechanical shocks, e.g., resulting from foot-ground impacts (Simonetti et al., 2018). According to Hussain et al., compliant actuators enable lightweight design with low endpoint impedance for wrist rehabilitation, while electromagnetic actuators are bulky and have high endpoint impedance (Hussain et al., 2020). Also, based on the study done by Chen et al., in 2016, compliant actuators are regarded as safe and human-friendly. These actuators are preferable over stiff actuators for various reasons. For example, such systems can deliver controlled force with back-drivability and low output impedance and are tolerant against shock and impacts. Chen et al. suggest using SEAs for assistive and rehabilitation robots (Chen et al., 2016).

On the other hand, passive robots must be equipped with passive actuators to enable scalable resistance and assistance based on the patient’s mobility status. There are various types of passive actuators such as friction brakes, viscous dampers, and elastic springs to name a few (Washabaugh et al., 2019).

Power transmission may be realized through the utilization of direct drive, gear, linkages, or cable-driven methods. Cable-driven transmission means allow for a more lightweight and compliant design. Backlash and transmission losses render the control of such systems challenging (Fischer et al., 2016). Sanjuan et al. classify cable-driven transmission into two categories of open-ended cables and closed-loop cables (Sanjuan et al., 2020). According to this study, open-ended cable systems exert forces in one direction, while close-ended cable systems exert friction forces (Sanjuan et al., 2020).

#### Sensing

In order to provide proper guidance for the device’s movement to execute the required tasks, the system should utilize sensing methods as input signals. Generally, four main sensors are used in rehabilitation devices, namely Motion and Position sensors, Force/Torque sensors, Electromyograms (EMG), and Electroencephalogram (EEG) (Shen et al., 2020). Since the rehabilitation devices are directly in contact with the human body, they should be reliable and highly accurate to provide the control system with real-time feedback of moving components (Zheng et al., 2005; Porciuncula et al., 2018).

The spatial configuration of the device is needed in order to analyze its kinematics and dynamics. For this purpose, position sensors are used to measure and indicate this spatial configuration. Among the sensors used for this purpose are: encoders, potentiometers, flex sensors, and transducers. For haptic applications like rehabilitation in VR, force/torque sensors are required. Usually, extra force/torque sensors are added to the system to provide additional safety levels. Gyro and acceleration sensors can be mounted on the mechanical structure for measuring the patient’s posture, for example, in HAL (Kawamoto et al., 2013).

Moreover, EMG and EEG could be used for measurements in noninvasive ways. The received signals in these two methods are often noisy and thus require further processing. Also, sensor fusion, in which the data from multiple sensors are merged, could provide a safer and more stable intention detection for the system.

#### Safety Measures

Last but not least, in the unsupervised context of home-based rehabilitation, safety is the most crucial criterion to be considered. Multiple redundant safety features must be incorporated in different modules of the device. In designing the mechanism stage, the device could be designed to offer intrinsic mechanical safety so as not to transfer excessive force to the patient’s body. Also, abnormal reactions of the patient should not be transferred to the actuator. For example, in Gloreha, these eventual reactions are absorbed by the mechanical transmission (Borboni et al., 2016). In “Design Issues for an Inherently Safe Robotic Rehabilitation Device,” Carbone et al. maintain that there are three major aspects that significantly affect the safety of the rehabilitation device, namely operation ranges, operation modes (speed, accelerations, paths), and operation force/torque (Carbone et al., 2018). Other measures could be taken to ensure safety; for example, Kim et al. utilized emergency stop buttons for both the user and the operator, hard stops at every joint, and also at the software level added safety features limiting the ROM and joint velocity and stopping the robot in case of excessive force/torque interaction (Kim and Deshpande, 2017). Feng et al. designed LLR-Ro to tackle the safety issue in three levels, i.e., mechanical limit, the electrical limit, and the software protection, to fully guarantee the limb safety of the patient in the whole training process (Feng et al., 2017). The device took advantage of inherent mechanical joint range of motion limits and limit switches placed on joint extreme positions to effectuate electrical limits and realizes the software protection according to the recorded patient information. Biomechanical, physiological, and even psychological information of patients can be utilized at software level safety checks to limit forces, motion, speed, and user adjustments to control parameters (Van der Loos and Reinkensmeyer, 2008).

Ultimately, at the end of each step, following the suit of the engineering design process, iterative optimization is needed to redefine the design process based on the predefined criteria. All of these design steps are highly interconnected and, therefore, should be considered from different aspects (Kruif et al., 2017). The HERO Glove was iteratively modified and redesigned based on evaluations and feedbacks from occupational therapists, specialized in stroke therapy, engineers, and stroke survivors (Yurkewich et al., 2019).

### Control System

After considering the mechatronic aspects, the control system has to be developed to ensure the proper behavior of the device (Desplenter et al., 2020). From the hierarchy point of view, it is suggested to group the control system into three levels, mission planning, trajectory planning, and state space control, where the state space control can itself be in two levels of supervisory control and low-level control. The mission planning level is responsible for task assignment and completion. Task assignment includes enabling the therapist or task planning algorithms on the therapy control unit to customize task details such as treatment strategy, initial angles and positions, movement period and dwell, movement velocities, motion patterns, number of repetitions, range of motion, to name a few. At this level, IoT technology can be utilized to provide proper feedback from the treatment and identification of muscloskeletal parameters for the therapist, as well as therapist supervision. To this end, a mission planning module must be developed proposing a wide variety of options applicable to the mechatronic design; including multiple modalities and training protocols can increase the chance of adoption by medical professionals (Desplenter and Trejos, 2020). According to the specified task, the trajectory planning level plans the kinematic and kinetic parameters of the motion to meet the objectives of the planned mission. For example, if the robot is supposed to provide a motion from an initial to a final configuration, the trajectory planning level designs the detailed variation of position, velocity and acceleration of the joints at each moment. If a force control mission is given, the force trajectory to be given to the control level is designed in the trajectory planning level. In the last level, the planned mechanical motion has to be implemented via the state space control level. Since the robot dynamic equations are normally nonlinear with unknown parameters and considerable uncertainties, a high-level supervisory control may be required to adaptively tune the control parameters recursively. This requirement can be handled easily by fuzzy logic or neural network schemes (Lin and Lee, 1991; Bai et al., 2018). The low-level control can then be a model-based control, where the computed torque method (CTM) is one of the most effective and convenient method for this level (Yıldırım, 2008).

During the last two decades, many solutions have been proposed to provide motion tasks suitable for post-stroke motor recovery. Priotteti et al. categorized existing global strategies for robotic-mediated rehabilitation into three groups of assistive, corrective, and resistive controllers (Proietti et al., 2016).

Passive assistance, where the device moves a muscle rigidly along the desired path by adopting different techniques, is the most common treatment strategy for acute patients due to the unresponsiveness of the paretic limb at early post-stroke stages. As soon as patients regain some degrees of mobilization, switching to other assistive solutions, such as triggered passive, where the patient initiates the assistance, or partial assistance, where the device assists the patient as needed (AAN), as utilized by Díaz et al. in HomoRehab (Díaz et al., 2018), may lead to better functional outcomes. Wai et al. provided a combination of the two mentioned assistive strategies in Ambidexter (Wai et al., 2018). Active initiation and execution of movements are the cornerstones of these strategies due to their proven effectiveness in stimulating neuroplasticity to enhance functional therapy.

The corrective mode is linked to the rehabilitation situation in which the patient is not performing the movement correctly, and the robot intervenes by forcing the impaired limb to the correct orthogonal direction. However, it is not easy to clearly differentiate assistive and corrective techniques. Hence, combined assistive-corrective controllers are mostly recurrent among existing controllers (Proietti et al., 2016).

When patients have recovered enough motor capacity, undergoing resistive therapies, which make tasks more difficult in some ways, may result in more significant rehabilitation gains (Ali et al., 2016). Added to the assistive controller, HomoRehab is also equipped with a resistive controller (Díaz et al., 2018). Resistive control, also termed as “challenge-based control,” can be achieved by merely applying constant resistance to the movement or error-based methods such as error augmenting and error amplification strategies (Ali et al., 2016). Existing devices mostly provide a combination of these three categories to offer a span variety of motion tasks adaptable to the patient’s degree of rehabilitation and the targeted impairment (Proietti et al., 2016). The control system of HAL exoskeletons is based on a hybrid control algorithm, consisting of two subsystems; one for voluntary control according to the patient’s voluntary muscles activity and another for autonomous control, which provides predefined physical passive assistance when patients are not able to generate required voluntary signals (Kawamoto et al., 2013). For asymmetrical gait rehabilitation, Curara^®^ employed a synchronization-based assistance control strategy using neural oscillators based on a central pattern generator network. The synchronization-based controller detects the user’s movement and measures the mutual gap between the user and the device, and then achieves a desired joint angle trajectory to be synchronized with human movements (Mizukami et al., 2018). Ren et al. proposed three different control strategies in their in-bed wearable ankle rehabilitation robotic device, namely triggered passive assistance for the early stages of recovery, and partial assistance and resistance when the patient gained some mobility (Ren et al., 2017). Their robot-guided rehabilitation protocol is capable of automatically switching between the control modes based on the patient situation and recovery status during the therapy.

Different control strategies can be realized by different methods and algorithms, such as position control, force control, force/position hybrid control, impedance/admittance control, or other control methods (Zhang et al., 2018). Due to their ability to provide natural, comfortable, and safe interaction between robots and patients, impedance control and its dual admittance control are two of the most common control algorithms currently used for rehabilitation exoskeletons, mostly for active training approaches (Proietti et al., 2016).

The difficult task of translating physiotherapist’s experience in manipulating the paretic limb into the desired trajectory for rehabilitative devices has made trajectory planning one of the issues for researchers in this field (Proietti et al., 2016). Proietti et al. summarized three main methods for defining reference trajectories, teach/record-and play (Pignolo et al., 2012), motion intention detection (Lyu et al., 2019), and optimization algorithms (Su et al., 2014).

Considering the type of human-machine interaction, control system inputs can be divided into bioelectric signals, e.g., EMG, and biomechanical signals, e.g., joint position, or a combination of these two types of signals (Desplenter et al., 2020). Biomechanical signals provide an accurate and stable interaction, especially for patients with high-level impairment, while biosignals are used for their potential in intention detection and neurological clinical applications due to their favorable effects on nerve rehabilitation treatment (Zhang et al., 2018).

Given the underlying significance of the control system in motor control recovery, some characteristics must be carefully practiced during design steps. Especially when designing for the home setting, providing an appropriately shared control between the device and the patient is of paramount importance. Such control enables a favorable autonomous therapy, including predictable behaviors for patients and never taking control when undesired by the user (Beckerle et al., 2017). It is also required for addressing neurorehabilitation issues not to suppress or hinder patients’ motor capabilities (JarrassÃ© et al., 2014).

Regarding the safety concerns of home therapy due to the absence of the therapist, highly adaptive control systems need to be developed to consider the differences of the human bodies, the motion tasks during the recovery process for each patient, and the nature of the injury between patients (Desplenter and Trejos, 2020). The control solution has to show an adequate amount of active compliance to avoid hurting the patient in the case of trajectory errors due to abnormal or excessive muscle contraction, spasticity, or other pathological synergies (Proietti et al., 2016). Impedance-based control schemes provide a dynamic relationship between force and position and could be utilized for adopting a safe, compliant, and flexible human-machine relationship. In this regard, LLR-Lo benefits from an amendment impedance control based on position control to realize motion compliance for patients in case of patient’s discomfort, which leads to aching movement of their leg while the mechanism leg is still moving (Feng et al., 2017). Analyzing the kinesthetic biomechanical capabilities of the target limb is also necessary for developing safe human-robot interaction (Atashzar et al., 2017). Compared to conventional therapy, relying on time or position-dependent trajectories can restrict rehabilitation therapy from the generalization of learning to new tasks. Thus, targeting goal-independent strategies would increase therapy’s efficacy, as patients are not constrained by following rigidly planned trajectories (Proietti et al., 2016).

As control system development tools, there are software frameworks designed to provide developers with a platform for the design and implementation of the control system, (e.g., Tekin et al., 2009; Coevoet et al., 2017; Desplenter and Trejos, 2020). The use of such frameworks supports the idea of standardization across different studies and devices. This enables a more efficient evolution and comparison of control systems, reduces the current ambiguity in classification, and neuters existing poor attention in documenting the control strategy of developed devices (Basteris et al., 2014), all assisting in identifying which strategy provides better results. WearMECS is a recently developed control software design and implementation tool available as an open-source software library. The framework provides a foundation for regulating the motion behavior of therapeutic mechatronic devices by considering three main control functionality groups, i.e., task-level control, estimation-level control, and actuation-level control (Desplenter and Trejos, 2020).

### User-Interfaces

The concept of remote supervision is entangled in home-rehabilitation. Developing a platform for tele-patient-doctor interaction is the key enabling factor for home-based therapy, in which the interaction of three parties of the patient, the therapist, and the device is provided (Desplenter et al., 2019). Harmonious completion of the rehabilitation program relies on the interaction between each of these parties, which takes place through two main user interfaces of the therapist and the patient. Therapists need to have access to the interface to prescribe treatment regimens and monitor patient’s progress in a scheduled plan through detailed reports containing the qualitative and quantitative evaluation of exercises. Patients also would have access to the prescriptions in their interface and move forward through the exercises. In the interface, the correct execution of motions should be displayed (Pereira et al., 2019). Before each exercise, visual and auditory descriptions need to be provided for the patient regarding how he/she is supposed to carry out the activity. The patient’s interface must provide feedback on the correctness of the performed activities, as well as a qualitative evaluation of his/her movement. All measurements and relevant data are collected, processed, and stored during each session and are remotely available. For example, in PAMAP the data is stored in the user’s electronic health record (EHR) (Martinez-Martin and Cazorla, 2019). In the end, IoT technologies can be utilized to connect the entire system together. These IoT-enabled software interfaces enable the therapist to remotely monitor a patient’s progress and tailor a customized treatment plan for him/her (Agyeman and Al-Mahmood, 2019).

Due to differences in patient demographics, needs, and characteristics of the target group must be carefully mapped to a set of general design principles. So as for considering the common cognitive impairments resulting from stroke or normal aging, there are certain factors to have in mind. These factors include ensuring clear directions, providing larger letters and numbering for improved readability, and avoiding vagueness, complexity, and involvement of what Egglestone et al. introduce as abstract thought (Egglestone et al., 2009; Cavuoto et al., 2018). Also, people with other pre-existing disabilities should also be taken into account, e.g., a text to speech component could be added to the interface for people with vision problems and sign language for people with hearing problems (Gomez-Donoso et al., 2017b).

To successfully replace the direct involvement of a therapist with a virtual one, the interfaces should provide options resembling those of traditional methods. Each interface has to be designed to replicate the traditional rehabilitation in a clinical setting, which means the therapist should be able to track the patient’s movements, assess his/her performance and analyze his/her progress to be able to prescribe the right treatment plan. Therefore, there is a need for careful evaluation of all of the steps that a therapist takes in a traditional setting to identify the parameters he needs to access. Once these parameters have been identified, user interface software through graphical interfaces (GUIs) provides access for displaying and editing stored data. This provides a base for therapists upon which they can make more educated decisions and choose the best treatment plan for their patients. Also, it is possible to add reminders to the system for notifying the patient to do his/her daily practices. In the end, the system uses this information to adapt the treatment program focusing on the parts that require more recovery to increase therapeutic value.

There are some other crucial principles to be considered for offering software interfaces that assist home-based systems in providing structured training sessions. A user-friendly interface in terms of environment and layout of features and components is the starting point of having a well-established interaction between patients and the device. This friendliness arises from patients’, as well as their caregivers’, ability to interact easily with the device in a familiar environment and to have quick and straightforward access to the training and monitoring components (Amirabdollahian et al., 2014). Enabling the patient to personalize the user interface environment, for example, in terms of shape or size of components or layout of different parts and modules, may increase the user-friendliness and create a sense of comfort and control.

For patients to be engaged and challenged during home rehabilitation in the physical absence of the therapist, there is a significant need for careful study and practice of motivational scenarios and features that ensure patient’s adherence to the treatment program. The most commonly identified and mentioned barriers to motivation are that rehabilitation scenarios are inherently repetitive and boring, having in mind that stroke patients themselves are prone to depression due to several factors such as social isolation and fatigue that is common among them (Egglestone et al., 2009; Borghese et al., 2013). The intrinsic entertaining characteristic of *Virtual/Augmented Reality (VR, AR)* and gaming present an alternative world in which mundane rehabilitation exercises are disguised in the shape of appealing fantasies. Integrating these methods into home rehabilitation is a common practice aimed at encouraging motivation in patients. The score-based nature of gaming further motivates patients—by rewarding points, coins, and badges as immediate feedback—and reinforces confidence and positivity through achievements (Hatem et al., 2016). VR, AR, and gaming offer plausible environments for versatile game design. Tuning the game content to the interests and needs of users (Egglestone et al., 2009), introducing variability and unpredictability to the tasks, e.g., by contextual interface concept, and programming real-world tasks by simulating real-life objects and events (Martinez-Martin and Cazorla, 2019), may encourage motivation even further. Another great feature of these methods, which makes them preferable to traditional ones, is that during therapy, the enjoyable experience distracts the patients from the prevalent potential pain induced by stroke (Gama et al., 2016). However, an important question remains regarding the efficacy and generalization of this virtual recovery to real world activities. Several studies have been conducted addressing this issue and found that training in a virtual environment offers equal, if not better, motor recovery compared to real-life training (Rose et al., 2005). According to Gama et al. (2016), AR might be more effective than VR, for it provides auto visualization during task execution and this, in turn, can improve patient corporal conscience and potentially engage additional visuospatial networks of the cortex; hence, it leads to more precision and accuracy respectively during interactive exercises and path traveling.

Designing games specifically for rehabilitation purposes, serious games, requires the incorporation of both entertaining and therapeutic goals. Games should offer fully customizable task options and hierarchical order of difficulty for each task. During the recovery process, patients’ mental and functional abilities improve, and tasks must be adapted to this variability to keep patients motivated by challenging yet achievable tasks at each stage of the recovery. In other words, by careful modification of the current skill level of the patient and challenge posed by the game, e.g., tuning assistance level and therapy intensity and difficulty, patients may go through a flow-like experience which in turn could potentially increase their active participation (Egglestone et al., 2009). In order to create a unified experience for the patient, it is preferable to develop games around a resonant theme, like Egglstone et al. approach, rather than unrelated minigames. In this approach, the systems do not merely offer video games but complete environments (Agyeman, Al-Mahmood, et al., 2019; Egglestone et al., 2009). It is also suggested to design flexible systems to have the capacity to integrate and support various devices. For example, audio-visual and haptic interfaces will enable the therapists to customize interaction mechanisms and to choose the proper device to deliver the best treatment regime with optimum efficacy for covering the wide range of disabilities, be it upper or lower. In this regard, Egglestone et al. developed a game that could be played by feet, hands, or whole body (Egglestone et al., 2009). In this way, a standardized game platform could be proposed to the entire community of therapists.

Training in home settings requires interface developers to design meaningful and consistent feedback, which should be similar to therapists’ clinical feedback—both on the quality of movement and their progress in performing tasks and achieving long-term and personal goals. Providing clear and customized feedback is another way of increasing engagement and motivation in patients, positively affecting retention and recovery (Kitago and Krakauer, 2013). Indeed, creating awareness in patients of their progress and offering a progress tracking module is one crucial approach toward stimulating internal motivation (Chen et al., 2019). Designing both immediate feedback during the therapy and after-session/long-term feedback must be taken into consideration as well. According to Guadagnoli et al., the immediacy of the feedback should be proportional to the task difficulty, i.e., the easier the task, the less frequent the feedback (Guadagnoli et al., 1996). Most current devices, as seen in the previous section, provided immediate visual, auditory, and haptic feedback to the patient (Lyu et al., 2019), which showed improved motivation, for example, by congratulation alarms after each correctly performed task, as well as educating and correcting task performances. Should the patient execute an exercise incorrectly, the system warns the patient and provides graphical help to guide the patient to perform it correctly. For instance, Gloreha provides a 3D simulated preview of the movement on the monitor as well as when the exercise is being performed (Bernocchi et al., 2018), PHAROS provides audio-visual descriptions (Martinez-Martin and Cazorla, 2019), and Lyu et al. generated haptic feedback through vibrating motors as punishment (Bernocchi et al., 2018; Lyu et al., 2019).

Daily and long-term summaries of patient records are another critical form of feedback, termed “knowledge of results” by Schmidt and Lee (1999), and it is a major source of motivation for patients. According to Chen et al. review study, a number of developers of home-based devices described how patients came motivated to proceed with therapy when they found themselves recovering (Chen et al., 2019). Progress tracking and visual feedback are better provided by graphical representation such as bar or pie charts, rather than numerical or analytical representation, to make a clearer sense of change at first glance. This can be particularly useful when the patients are mostly among elderlies, and it may be difficult for them to analyze the results. Note that patients’ dependence on feedback is not favorable, and to avoid that, it is suggested to lower the feedback frequency over time (Kitago and Krakauer, 2013).

When designing for home use, particularly due to the absence of a medical professional, it is essential to consider therapeutic and clinical requirements from the therapists’ perspective. Based on the premise that adoption of mechatronic rehabilitation devices heavily relies on the acceptability of the device from the therapist’s point of view, Despleneter et al.surveyed Canadian therapists to extract design features that successfully convey the needs of the therapists. Then they mapped the derived data into software requirements for an enhanced therapist-device relationship (Desplenter et al., 2019). A good therapist-device relationship is desirable for equipping therapists with an extensive data set of patient history to be assessed and analyzed toward optimized and educated planning of treatment strategies. Participants mentioned the five areas of patient history, pain, motion, activities, or strength as their most favorable data to be tracked over time for patients’ assessment. Desplenter et al. concluded that the designed software system should have features such as standard data records, treatment plan templates, and patient evaluation scales. The user should also be able to design and customize these templates and scales to meet the needs of a particular treatment strategy, both qualitative and quantitative. For this purpose, time-stamped data should be collected, and numerical analyses and visualizations should be generated based on the quantitative data to provide reports. Visualization, particularly in graphs and tables, facilitates data assessment, and puts them into perspective for the therapists. Eventually, a cumulative report must be provided in the therapist’s interface in which session history, the patient’s history, and progress tracker are among its most important components.

## Conceptual Framework

In the previous sections, some of the existing home-based rehabilitation devices were briefly reviewed, and after a general analysis of current challenges and shortcomings, a guideline for designing the components of these systems, in particular for utilizing new advanced technologies, was discussed. As described before, there is a need for a telerehabilitation platform that concerns remote supervision implementation by interconnecting all described modules. To this end, reviewed home-based systems have leveraged different approaches. For example, HomeRehab benefited from a cloud service for exchanging performance and therapy information between the patient and the therapist (Díaz et al., 2018). Amirabdollahian et al. utilized a tele-robotic support platform in their SCRIPT project consisting of a replicated database, a healthcare professional web portal, and a decision support system (Amirabdollahian et al., 2014). Chen et al. also benefited from a web server for the Tele-Doctor-Patient Interaction module in their proposed rehabilitation wheelchair system (Chen S. et al., 2017). Ambidexter provides remote rehabilitation through IoT technologies (Wai et al., 2018). While the aforementioned systems undoubtedly help with remote supervision and patient-therapist interaction, most of them are case-specific for their device and do not offer an interoperable platform allowing for supporting other devices. On the other hand, telehealth platforms for in-home interventions, which need more supervision, information exchange, and online communication, have gained significant traction over the recent years, (e.g., Wang et al., 2017; Kouris et al., 2018; Hilty et al., 2019; Tun et al., 2020; Tsiouris et al., 2020). Among existing telehealth frameworks, they either do not specifically and comprehensively address post-stroke robotic rehabilitation interventions or do not cover the beyond-physical-rehabilitation demands of this population. Therefore, to address the aforementioned limitations as well as to cover the real-life needs of post-stroke individuals, we propose a versatile conceptual framework for community-based robotic in-home rehabilitation. Our proposed framework conceptually provides a unified interoperable solution for interconnecting various rehabilitation robots, lays a foundation for in-home rehabilitation and supports beyond-rehabilitation needs of the post-stroke community—like the need for socialization and assisting in gaining independence for daily activities like shopping, especially during pandemics. Our framework offers a comprehensive solution addressing the multi-faceted needs of involved parties in post-stroke rehabilitation, including patients, healthcare professionals and researchers. To this end, some existing telehealth frameworks, e.g., HOLOBALANCE (Tsiouris et al., 2020), could potentially be modified and adapted for implementing our conceptual framework.

**FIGURE 6:**
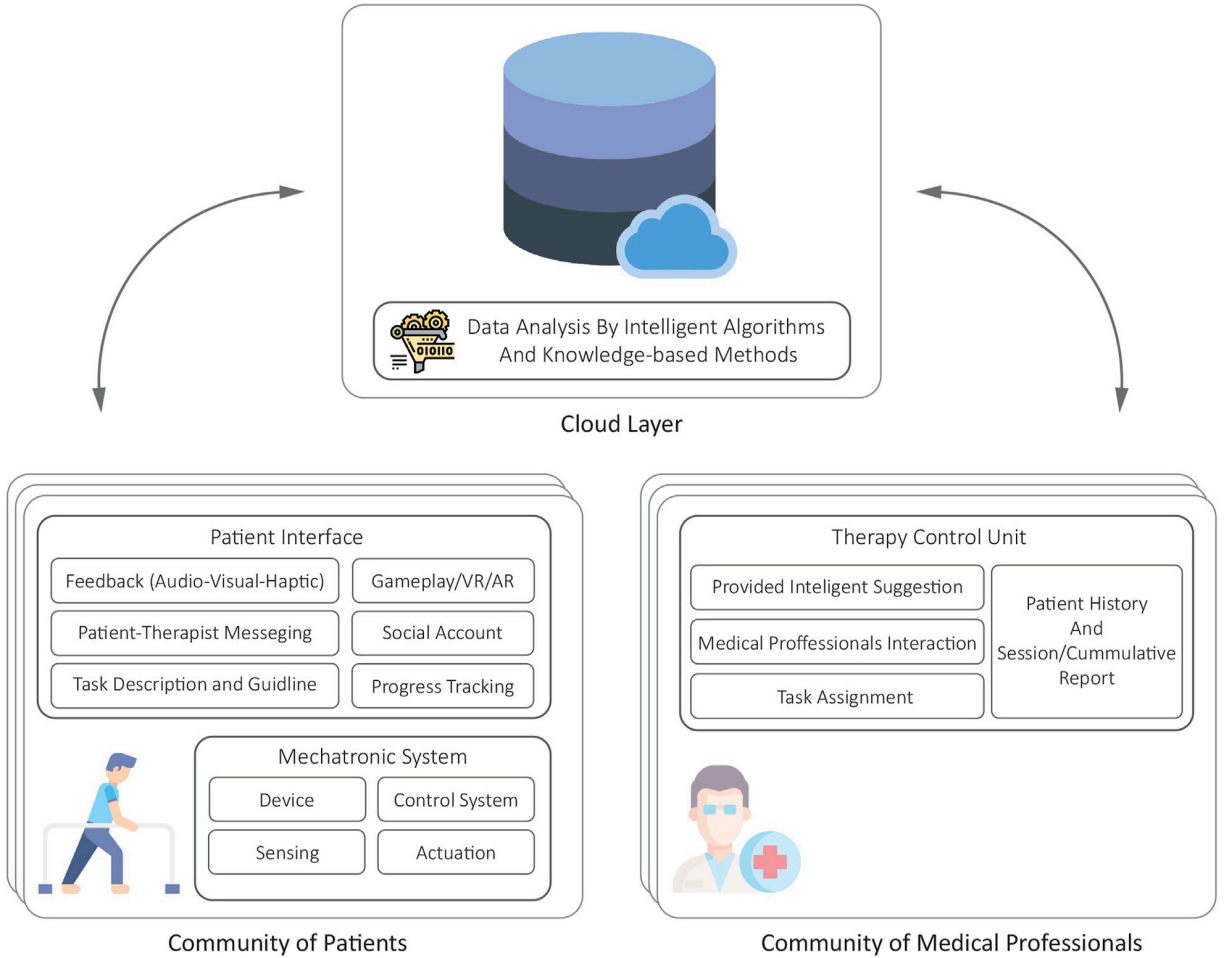
The proposed conceptual framework for community-based home rehabilitation.

This framework’s central concept is based on the development of a multi-agent IoT communication system in which patients, therapists, and administrators each represent an agent of their community interacting with each other over the provided platform (Figure 6). This interaction utilizes cloud-based methods for computation, storage and analysis of data collected from each agent. The acquired data is initially processed before being transmitted to cloud and fog servers, where they form a large data set. According to Sahu et al. (2020), due to the sensitive nature of medical data, security, privacy, and confidentiality, as well as the integrity of these data, are of critical importance. Hence, to monitor these concerns, precautionary safety measures must be developed in advance. Also, policies and rules, defining and authorizing the access level of each agent, need to be carefully established and implemented in order to ensure and enhance the security and privacy of the system (Sahu et al., 2020).

The acquired data can be initially processed before being transmitted to cloud and fog servers, where they form a large data set. For example, online identification of musculoskeletal parameters and their variation during treatment can be conducted and plotted in the smartphone, PC, or any other digital processor which is connected to the rehabilitation robot. These preprocessing features can be based on either model-based approaches, or artificial intelligence (AI) algorithms such as artificial neural networks. Although training of such systems can be computationally very demanding for these processors, the training process is performed mostly offline. Applying the trained AI feature and even its required recursive update does not involve significant computation, and can be easily handled on the processor of a smartphone, PC, or any other available digital processor.

Acquired data from agents should be comparable with each other, and therefore it is critical to bring the collected data into a common format which occurs through data standardization. By providing internally consistent data with the same content and format, any confusion and ambiguity are avoided. This ensures that all parties involved have mutual understanding, and view the results in the same way which leads to reliable measurements and decision plans. Standardized quantitative and qualitative data should be collected for reliable and educated assessment of patients’ performance through evaluation forms and outcome measurements. From a therapist’s point of view, the prescribed tasks, reports, and evaluations should be offered in a standard format. To do so, there needs to be a predetermined data extraction on which the community of healthcare professionals has reached a consensus. However, in case of any ambiguity or to provide further details and explanations, therapists should have the option of attaching notes to the data (Desplenter et al., 2019).

The proposed community-based rehabilitation framework facilitates knowledge sharing and provides comprehensive data to ensure educated judgment regarding the best treatment regimen yielding the best recovery outcome. This massive pool of information—both from recorded data and also normalized datasets from already existing medical center repositories—generates big data. By applying machine learning, meaningful information from these large data sets can be extracted. This extensive database can be beneficial for various applications, one of which could be providing a research base enabling further studies in fields of neurorehabilitation, engineering, and occupational/physical therapists. Thus, in this IoT-enabled rehabilitation system, data analysis is performed by intelligent algorithms and knowledge-based methods to provide intelligent suggestions to the therapists, creating a basis for the best treatment plan assignment.

In the framework, the capability of communication among agents of each community is also feasibly provided. The interaction of healthcare professionals gives them the opportunity to clarify any issues or ambiguities, gain additional expertise, discuss possible treatments, and share their knowledge. The provided platform can also enable socialization among patients. Social restrictions imposed by stroke and exacerbated by the COVID-19 pandemic, have adversely affected patients’ lives. Therefore, any means of social interaction could potentially improve their quality of life, which in turn could further motivate them to adhere to the therapy (Egglestone et al., 2009). This community-based rehabilitation allows the possibility of performing rehabilitation in the connected groups, for example, through multiplayer gaming. By optional assignment of a social media profile to each patient ID, they could also share their achievements and support each other.

We suggest an alternative approach in which the purpose of the framework goes beyond rehabilitation to contribution to society and household chores. The virtual environment tasks should be designed so that, along with rehabilitation, they can offer patients the opportunity to learn real-life and daily living skills—cooking, gardening, and housekeeping, to name a few. For example, learning the cooking skill takes place in a virtual kitchen where all of the ingredients and utensils are available along with cooking instructions in the form of video and audio to enable the patient to learn and practice simultaneously. The theme-based free world gameplay would be one of the best options as it can be flexible enough to reflect various aspects of daily life. For example, Saebo Inc. has developed a virtual reality environment in which in the cooking scenario, should the virtual kitchen lack any of the ingredients or utensils required to complete the task, the game enables the patient to go grocery shopping; this way through a comprehensive experience, patient not only goes through therapy but gets to enhance his/her life skills. Integrating this free world environment with our suggested community-based framework adds an additional social layer where patients can teach and learn these skills from each other. For instance, if a patient knows a particular recipe, she/he can teach and share it with the patient community. This provides the patients with a sense of control over their treatment and fosters a sense of usefulness, purpose, and contribution to society. Also, in this time of COVID-19, where everybody, especially elders, feels disconnected from the world, this will excite the feelings of inclusion and connection, which in turn will further motivate the patients to partake more frequently and actively in their treatment sessions.

To go one step further, this virtual environment could be linked to existing online shops allowing the patient to contribute to household chores by purchasing the required and essential items for the house and facilitating the acquisition of his/her own necessities. According to Brenner and Clarke (2019), shopping difficulty is one of the most common self-reported difficulties with daily activities among individuals with disabilities. Thus, the enhanced autonomy and independence in patients alleviates the prevalent feeling of being a burden to their family which in turn promotes a sense of achievement and self-worth. The suggested alternative approaches and rehabilitation are not mutually exclusive, as maintaining engagement in daily life activities is critical for functional and motor recovery (Brenner and Clarke, 2019). As of 2020, due to restrictions caused by the COVID-19 Pandemic and the importance of adhering to social distancing measures, online shopping has become ubiquitous, making this option ever-increasingly plausible.

Finally, the framework has the capacity to incorporate an ambient assisted living ecosystem that can provide patients, specifically elders, with personalized options. These options include remote monitoring, assessment, and support based on their unique profiles and surrounding context. The purpose of such options is to enhance the independence of elderly or disabled individuals in their own secure and convenient space of living (Al-Shaqi et al., 2016; Sahu et al., 2020; Stavropoulos et al., 2020).

Rehabilitation programs, even in the clinical setting, are often not fully supported by public health systems, and hence not all post-stroke patients can afford this option, and due to the enormous socioeconomic impact and high costs, not just on the patient but also on their families, patients opt to drop out (Borghese et al., 2014). Additionally, implementing the proposed framework for post-stroke telerehabilitation, imposes extra costs to the patients for providing the required hardware and software basis of the telehealth framework. For home-based rehabilitation to be acceptable by all end-users involved (patients, clinicians, therapists, and health administrators), these challenges and limitations need to be addressed both by healthcare providers and technology developers. Burden and strain potentially imposed on caregivers are other limiting concerns. However, implementation of the proposed telerehabilitation framework could offer valuable functional outcomes for motor recovery of the wide range of the post-stroke population. Future work should focus on evaluating the proposed telerehabilitation framework for community-based remote therapy during and beyond the pandemic, in terms of different use dimensions, technological acceptance by the stakeholders, clinical efficacy of the system and its economic efficiency.

## Conclusion

Early-stage post-stroke rehabilitation, a crucial phase of rehabilitation, could be a direct indicator of the possibility of motor recovery. In the current restrictive climate of the COVID-19, due to the restrictions associated with social distancing and limitations for commuting, it has become more difficult for patients to use this sensitive window of time for rehabilitation treatments. In this context, home-based rehabilitation can be a solution.

Several home-based rehabilitation devices have been developed in recent years, both at the research and commercial levels. Although there are some challenges in the research and development of these devices, based on a technical evaluation of aspects such as mechatronics, the control system, and software, these devices can be tuned to be suitable for home-based rehabilitation therapy. Eventually, such systems can be utilized in a framework aiming at creating a comprehensive network for therapists, patients, engineers, and researchers. To this end, standardization in every aspect of home-based systems, from design criteria to performance evaluation, is required.

The COVID-19 pandemic has proved the necessity of further deploying the power of the internet and computers, not only for the purpose of communication but also for big data analysis. Such technologies can be feasibly integrated with mechatronic systems such as rehabilitation robots to enable new applications such as remote home-based therapies. The outcome of such an interconnected framework would be significantly vital for the recovery of post-stroke patients, promoting the quality of their lives, and eventually reducing the associated burdens on the healthcare system in the long term.

## Data Availability

The original contributions presented in the study are included in the article/Supplementary Material, further inquiries can be directed to the corresponding author.

## Glossary

MAL: Motor activity log
MAL QOM: Motor activity log (quality of movement)
BBS: Berg balance scale
FMAS: Fugl-Meyer assessment scale
FMUE or FMA-UE: The Fugl-Meyer assessment for motor recovery after stroke for the upper extremity
FMA-Hand: The Fugl-Meyer assessment for hand
FMLE or FM-LE: The Fugl-Meyer assessment for motor recovery after stroke for the lower extremity
FM: Fugl-Meyer assessment of motor recovery
TUGT: Timed up and go test
SPPB: Short physical performance battery
DGI: Dynamic Gait index
mRS: Modified Rankin scale
CMSA-H: Chedoke McMaster stroke assessment stage
FES(S): Falls-efficacy scale Swedish version
BI: Barthel index
FIM: Functional independence measure
ARAT: Action research arm test
WMFT: Wolf motor function test
GWMFT or GWMFT-func: The graded wolf motor function test
GWMFT-time: Graded wolf motor function test (completion time)
CAHAI: The Chedoke arm and hand inventory
MAS: The motor assessment scale
MMAS: The modified Modified ashworth scale
EXT: Finger extension force
GS: Grip strength
LPS: Lateral pinch strength
PPS: Palmar pinch strength
MTS: Modified Tardieu scale
SIS: Stroke impact scale
SUS: System usability scale
BBT: Box and blocks test
JTHFT: Jebsen-Taylor hand function test
10MWT: 10 minute walk test
6MWT: 6 minute walk test
K-FES: Korean falls-efficiency scale
FE: Flexion/Extension
F: Flexion
E: Extension
PS: Pronation/Supination
IE: Inversion/Eversion
PD: Plantarflexion/Dorsiflexion
